# ICAP‐1 loss impairs CD8^+^ thymocyte development and leads to reduced marginal zone B cells in mice

**DOI:** 10.1002/eji.202149560

**Published:** 2022-05-13

**Authors:** Silvia Sevilla‐Movilla, Patricia Fuentes, Yaiza Rodríguez‐García, Nohemi Arellano‐Sánchez, Peter W. Krenn, Soledad Isern de Val, Sara Montero‐Herradón, Javier García‐Ceca, Valeria Burdiel‐Herencia, Sofía R. Gardeta, Noemí Aguilera‐Montilla, Celia Barrio‐Alonso, Georgiana Crainiciuc, Daniel Bouvard, Angeles García‐Pardo, Agustin G. Zapata, Andrés Hidalgo, Reinhard Fässler, Yolanda R. Carrasco, Maria L. Toribio, Joaquin Teixidó

**Affiliations:** ^1^ Department of Molecular Biomedicine Centro de Investigaciones Biológicas Margarita Salas (CSIC) Madrid Spain; ^2^ Development and Function of the Immune System Unit, Centro de Biología Molecular Severo Ochoa, CSIC Universidad Autónoma de Madrid Madrid Spain; ^3^ Department of Molecular Medicine Max Planck Institute of Biochemistry Martinsried Germany; ^4^ Department of Biosciences and Medical Biology, Cancer Cluster Salzburg Paris‐Lodron University of Salzburg Salzburg Austria; ^5^ Department of Cell Biology, Faculty of Biology Complutense University of Madrid Madrid; ^6^ Spain and Health Research Institute Hospital 12 de Octubre (imas12) Madrid Spain; ^7^ Department on Immunology and Oncology Centro Nacional de Biotecnología (CNB)‐CSIC Madrid Spain; ^8^ Centre de Recherche en Biologie Cellulaire de Montpellier Montpellier France; ^9^ Institute for Cardiovascular Prevention Ludwig‐Maximilians University Munich 80336 Germany; ^10^ Hospital General Universitario Gregorio Marañón Madrid Spain; ^11^ Area of Developmental and Cell Biology Centro Nacional de Investigaciones Cardiovasculares Carlos III (CNIC) Madrid Spain

**Keywords:** B‐ cell maturation, cell adhesion, ICAP‐1, integrins, thymocyte development

## Abstract

ICAP‐1 regulates β1‐integrin activation and cell adhesion. Here, we used ICAP‐1‐null mice to study ICAP‐1 potential involvement during immune cell development and function. Integrin α4β1‐dependent adhesion was comparable between ICAP‐1‐null and control thymocytes, but lack of ICAP‐1 caused a defective single‐positive (SP) CD8^+^ cell generation, thus, unveiling an ICAP‐1 involvement in SP thymocyte development. ICAP‐1 bears a nuclear localization signal and we found it displayed a strong nuclear distribution in thymocytes. Interestingly, there was a direct correlation between the lack of ICAP‐1 and reduced levels in SP CD8^+^ thymocytes of Runx3, a transcription factor required for CD8^+^ thymocyte generation. In the spleen, ICAP‐1 was found evenly distributed between cytoplasm and nuclear fractions, and ICAP‐1^–/–^ spleen T and B cells displayed upregulation of α4β1‐mediated adhesion, indicating that ICAP‐1 negatively controls their attachment. Furthermore, CD3^+^‐ and CD19^+^‐selected spleen cells from ICAP‐1‐null mice showed reduced proliferation in response to T‐ and B‐cell stimuli, respectively. Finally, loss of ICAP‐1 caused a remarkable decrease in marginal zone B‐ cell frequencies and a moderate increase in follicular B cells. Together, these data unravel an ICAP‐1 involvement in the generation of SP CD8^+^ thymocytes and in the control of marginal zone B‐cell numbers.

## Graphical abstract

Graphical Abstract: Lack of ICAP‐1 leads to impaired single‐positive CD8^+^ thymocyte development without affecting integrin α4β1‐dependent thymocyte adhesion. ICAP‐1 showed a strong nuclear distribution in thymocytes. ICAP‐1^–/–^ spleen T and B cells displayed upregulation of α4β1‐mediated adhesion and reduced proliferation. Loss of ICAP‐1 led to decreased marginal zone B‐cell frequencies.

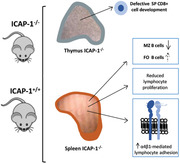

## Introduction

Integrins are heterodimeric cell membrane proteins composed of noncovalently associated α‐ and β‐subunits which mediate cell adhesion [1]. The cytoplasmic domains of the β‐subunits of integrins contain binding sites for proteins which regulate integrin activation and cell adhesion including talin, kindling, and ICAP‐1 [2, 3]. Talin binds to membrane‐proximal NPXY and kindlin to membrane‐distal NPXY motifs in β‐subunit cytoplasmic domains, causing an extension of the heterodimeric ectodomain from a bent inactive conformation, and leading to high‐affinity, active integrins supporting cell adhesion [2, 4, 5].

The ICAP‐1 large isoform, also known as ICAP‐1α or ITGB1BP1, is ubiquitously expressed and contains a C‐terminal phosphotyrosine‐binding domain that interacts with a valine residue located at position‐5 from the membrane‐distal NPXY β1 motif [6, 7, 8]. ICAP‐1 can interact with β1, but not with β2, β3, or β5 cytoplasmic regions [6, 7]. By competing with talin and kindlin for binding to β‐subunit cytoplasmic regions, ICAP‐1 can suppress integrin activation [9, 10]. ICAP‐1 also contains an *N*‐terminal nuclear localization signal (KKRH) enabling its nuclear translocation [11, 12, 13], suggesting that ICAP‐1 might have integrin‐dependent and ‐independent functions. Located downstream of this nuclear localization signal resides a motif susceptible of Ser and Thr phosphorylation, with potential to regulate integrin activation and ICAP‐1 cellular distribution [12, 13, 14]. In addition to bind β1, ICAP‐1 can also interact with KRIT1 (Krev interaction trapped‐1; also known as CCM1), a protein containing three NPX(Y/F) motifs, of which the first one mediates interaction with ICAP‐1 [15, 16]. KRIT binding to ICAP‐1 blocks ICAP‐1 interaction with β1, leading to upregulated integrin activation [12, 16, 17].

Characterization of ICAP‐1‐null mice revealed defective osteoblastic function and showed that ICAP‐1‐deficient osteoblasts harbor integrin receptors, with an active conformation leading to increased adhesion to fibronectin [18]. Furthermore, ICAP‐1 was shown to play important roles in the development of the vascular system [17]. In addition, we recently reported that ICAP‐1‐silenced multiple myeloma cells display upregulated adhesion to the α4β1 integrin ligands VCAM‐1 and the CS‐1 region of fibronectin (CS‐1/FN) [19].

Immune cells and BM HSPCs express several β1‐class integrins [20], especially α4β1. This integrin mediates important interactions of HSPC and B cells with their ligands in the BM microenvironment [21, 22], is involved in cell trafficking to sites of inflammation, and contributes to lymphocyte migration to lymphoid tissues [23]. Given that ICAP‐1 has the potential of competing with talin and kindlin for β1‐integrin binding, thus, being able to regulate cell adhesion, and in light of its potential integrin‐independent functions, we hypothesized that ICAP‐1 might regulate the immune cell function. In the present work, we tested this hypothesis using ICAP‐1‐KO mice.

## Results

### Normal hematopoietic stem and progenitor cell content and unaltered B‐ lymphopoiesis in ICAP‐1^–/–^ mice

**Figure 1:**
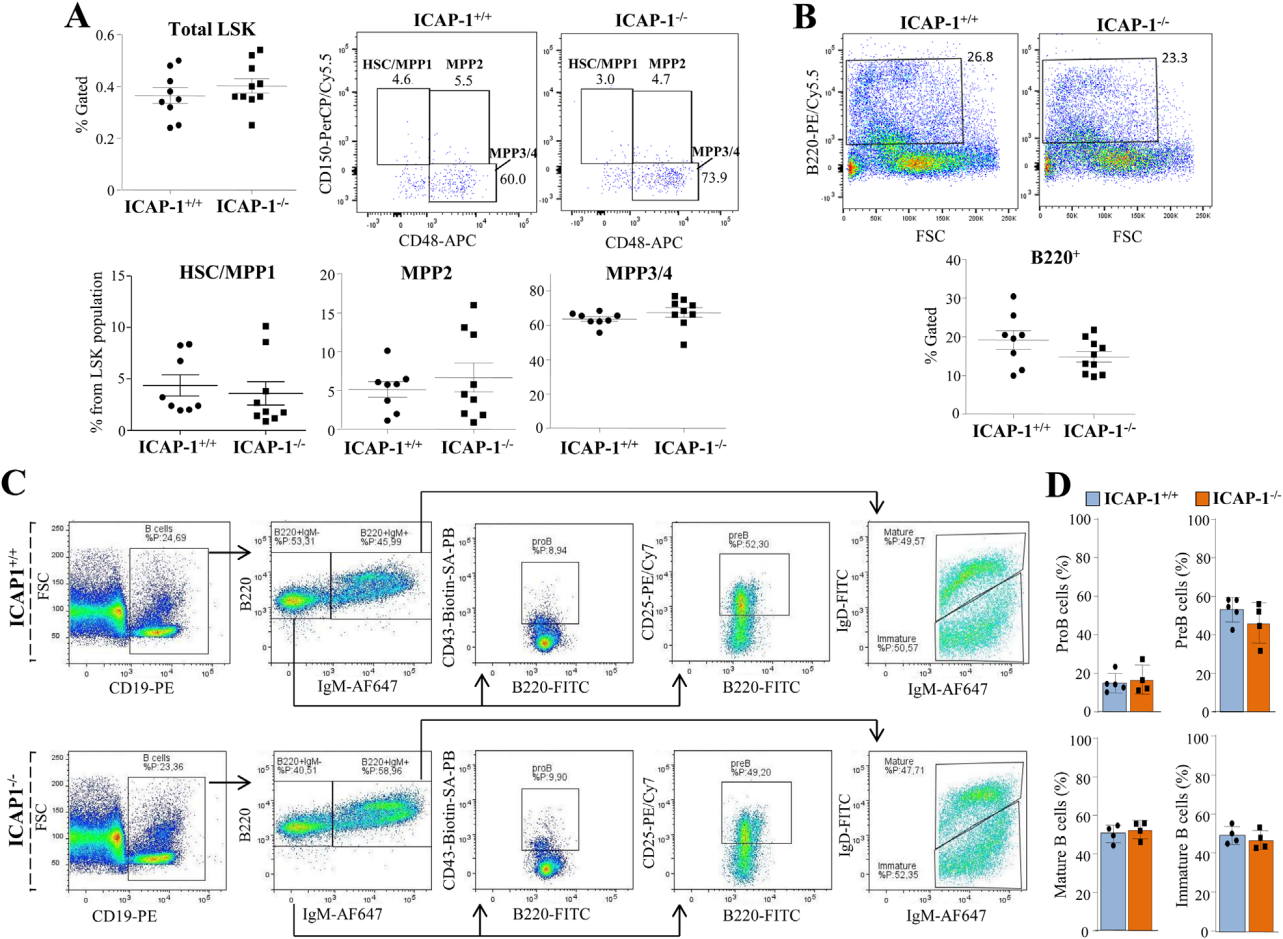
**Analysis of BM cell populations in ICAP‐1‐null mice**. (A) LSK cells (from total BM), and HSC/MMP1, MPP2 and MPP3/4 BM cell populations from ICAP‐1^+/+^ and ICAP‐1^–/–^ mice were analyzed by FACS. (B) Percentage of BM B220^+^ cells was assessed by FACS. (C) Gated CD19^+^ BM cells were subsequently gated on B220^+^ cells as indicated (either PE‐ or FITC‐labelled depending on the posterior antibody mixtures), and analyzed for the expression of CD43 and CD25, and IgM and IgD. (D) Data quantification of the indicated B‐cell populations in the BM of ICAP‐1^+/+^ and ICAP‐1^–/–^ mice. Panels A and B show representative dot plots and pooled data from three independent experiments. Panels C and D display representative dot plots and pooled data from two independent experiments.

Confirming earlier results [18], ICAP‐1^–/–^ mice were smaller than control animals, which was especially evident among 3‐ to 5‐week old littermates (Supporting information Fig. S1A). We did not find significant differences in cell numbers from BM samples relative to body weight of control and ICAP‐1‐null mice (Supporting information Fig. S1B). *ICAP‐1* is expressed in BM hematopoietic stem and progenitor cells (HSPCs) (Gene Expression Commons, http://gexc.stanford.edu, and GEO GSE77078) [24]. To study if ICAP‐1 loss affects HSPC populations in BM, committed cells were first gated out from subsequent FACS analyses, thereby focusing on lineage negative cells harboring the Sca‐1^+^c‐kit^+^ (LSK) markers. There were no remarkable differences in LSK cells between ICAP‐1^+/+^ and ICAP‐1^–/–^ mice (Fig. 1A, top left; and Supporting information Fig. S1C). Further analysis using the CD150 and CD48 markers showed no major changes in HSC/multipotent progenitor cell 1 (MPP1; LSK CD48^–^ CD150^+^), MPP2 (LSK CD48^+^ CD150^+^), or MPP3/4 (LSK CD48^+^ CD150^–^) (Fig. 1A, top middle and right, and bottom panels). These results reveal that ICAP‐1 does not contribute to the HSPC compartment at any stage of their differentiation program.

The frequency of B220^+^ BM B cells was also similar in control and ICAP‐1^–/–^ mice (Fig. 1B), and we found no significant alterations in cell numbers and proportions in the B220^+^IgM^–^ and B220^+^IgM^+^ fractions in ICAP‐1‐null mice (Fig. 1C; Supporting information Fig. S1D). Analysis of B‐cell progenitors from the proB stage (CD19^+^‐gated), and based on B220/CD43 and B220/CD25 expression, revealed no differences in the proB and preB cell distribution, and we also found no remarkable changes in immature and recirculating mature B cells, according to IgM and IgD expression levels, respectively (Fig. 1C and D). These results suggest that ICAP‐1 is dispensable (or redundant) for medullary B lymphopoiesis.

### Defective CD8^+^ single‐positive thymocyte development in ICAP‐1‐null mice

ICAP‐1 expression was found in thymic cells, largely composed of thymocytes (Supporting information Fig. S1E, top). In agreement with a proportionate dwarfism, ICAP‐1^–/–^ thymi were smaller than those from control mice (Supporting information Fig. S1E, bottom), and displayed a reduced total cellularity, although no remarkable differences in cell numbers per body weight were observed between control and ICAP‐1^–/–^ thymi (Supporting information Fig. S1F).

**Figure 2:**
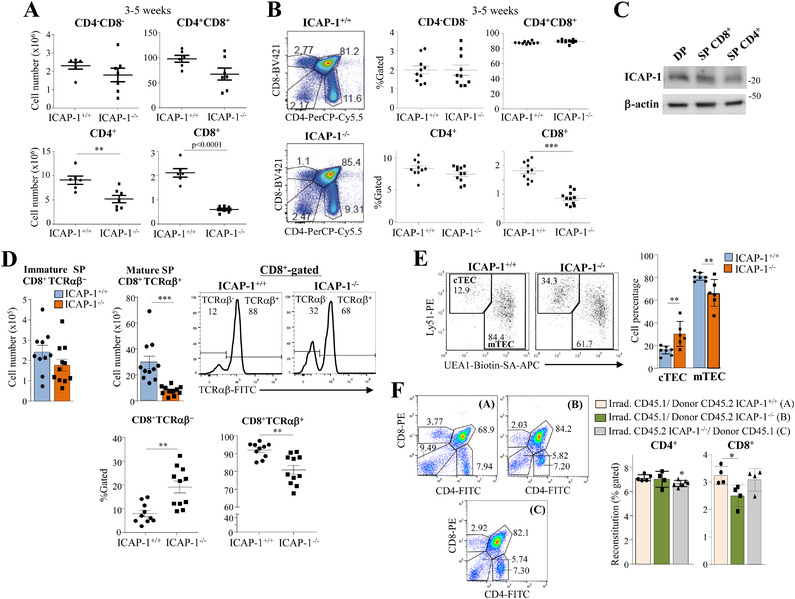
**Defective SP CD8^+^ thymocyte development in ICAP‐1‐null mice**. Total numbers (A) and proportions (B) of the indicated thymocyte subsets in ICAP‐1^+/+^ and ICAP‐1^–/–^ mice were analyzed by flow cytometry. (C) Expression of ICAP‐1 in the isolated thymocyte populations was analyzed by immunoblotting. Loading control was assessed with anti‐β‐actin antibodies. Shown is a representative result out of two independent experiments. (D) Cell numbers and frequencies of the indicated immature and mature SP CD8^+^ thymocyte subpopulations were determined by flow cytometry. (E) Dot plots (left) and proportions (right) of EpCAM^+^CD45^–^‐gated cTEC and mTEC subpopulations. (F) Thymi from the indicated chimeric mice were analyzed by FACS to assess percentages of SP CD4^+^ and CD8^+^ cells. A, B, D, and E show pooled data from at least three independent experiments. Representative dot plots and histograms are shown in panels B, D, E and F (^***^ *p *< 0.001; ^**^ *p *< 0.01; ^*^ *p* < 0.05).

Analysis of thymocytes at the CD4^–^CD8^–^ double‐negative (DN) developmental state revealed that the total numbers and proportions of DN cells were not significantly altered in ICAP‐1‐deficient mice (Fig. 2A and B; Supporting information Fig. S2A and B). Although, we found a small decrease in cell number and frequency of the DN1 (CD44^+^CD25^–^) thymocyte subset in ICAP‐1^–/–^ thymi relative to control ones, it did not reach statistical significance, whereas the proportions of the DN2 (CD44^+^CD25^+^), DN3 (CD44^lo^CD25^+^), and DN4 (CD44^–^CD25^–^) subsets were comparable to the controls (Supporting information Fig. S2C and D). These results indicate that lack of ICAP‐1 does not substantially affect thymic T‐cell development at the DN stage.

Reduced numbers of CD4^+^CD8^+^ double‐positive (DP), and CD4^+^ and CD8^+^ single‐positive (SP) thymocytes were found in ICAP‐1^–/–^ mice, especially in 3‐ to 5‐week‐ old animals (Fig. 2A; Supporting information Fig. S2A). However, the frequencies of DP and SP CD4^+^ cells were unaffected in ICAP‐1‐null thymi, whereas those from SP CD8^+^ thymocytes were significantly lower in ICAP‐1^–/–^ than in control thymi (Fig. 2B; Supporting information Fig. S2B), in spite that ICAP‐1 is expressed at similar levels in DP and SP CD4^+^ and CD8^+^ thymocytes (Fig. 2C **)**.

CD8^+^ SP thymocytes encompass both immature SP (ISP) TCRαβ^−^CD8^+^ and mature SP TCRαβ^+^CD8^+^ subpopulations during murine T‐cell development [25]. Analysis of the impact of ICAP‐1 deficiency on the distribution of both SP CD8^+^ subsets revealed a significant decrease in absolute numbers and frequencies of the mature SP subset in ICAP‐1‐null relative to control counterparts, whereas no alterations were detected in ICAP‐1^–/–^ ISP cell numbers, with this subset displaying a higher cell proportion than the control ISP subpopulation (Fig. 2D). These results reveal a deficient generation of mature SP TCRαβ^+^CD8^+^ thymocytes in mice lacking ICAP‐1.

### Defective CD8^+^ SP cell development in ICAP‐1^–/–^ mice is thymocyte‐autonomous

Isolated EpCAM^+^CD45^–^ thymic epithelial cells (TECs) displayed low *Icap‐1* levels (Supporting information Fig. S3A). The distribution of Ly51^+^UEA1^–^ cortical (c) and Ly51^–^UEA1^+^ medullary (m) subsets in EpCAM^+^CD45^–^ TEC was analyzed by flow cytometry. Data showed a moderate increase in the frequency of cTECs linked to reduced proportions of mTECs in ICAP‐1^–/–^ thymi as compared with ICAP‐1^+/+^ counterparts (Fig. 2E), indicating that loss of ICAP‐1 causes a partial defect of mTEC generation. Examination of ICAP‐1^–/–^ thymi sections revealed no major alterations in the distribution of DP cells in the PanCK^+^ thymic cortex, or in the distribution of the SP CD4^+^ and CD8^+^ thymocytes in the medullary K5^+^ area (Supporting information Fig. S3B).

To investigate whether the deficient SP CD8^+^ cell development in ICAP‐1^–/–^ mice was thymocyte‐ or TEC‐intrinsic, we first inoculated equal numbers of CD45.2^+^ ICAP‐1^+/+^ and ICAP‐1^–/–^ BM cells into the same lethally irradiated CD45.1^+^ B6.SJL hosts, allowing both cell types to develop in an identical environment. These experiments revealed that ICAP‐1^–/–^ BM cells are completely outcompeted by the ICAP‐1^+/+^ hematopoietic counterparts (data not shown), and therefore, we generated single BM chimeric mice. In line with the decreased proportion of SP CD8^+^ thymocytes in ICAP‐1^–/–^ mice, lethally irradiated CD45.1^+^ mice transplanted with BM from ICAP‐1‐null animals displayed a significant 20–25% reduction in thymus SP CD8^+^ cells relative to the irradiated mice reconstituted with BM from ICAP‐1^+/+^ animals (Fig. 2F; Supporting information Fig. S3C and D). Instead, the proportion of thymic SP CD4^+^ cells in the reconstituted mice did not change. When irradiated CD45.2^+^ ICAP‐1^–/–^ mice were transplanted with BM from control CD45.1 animals, we did not detect alterations in CD8^+^ SP frequencies compared to control irradiated animals, although a 5–8% decrease in SP CD4^+^ cells was observed (Fig. 2F; Supporting information Fig. S3C and D). Together, these results indicate that the deficient SP CD8^+^ cell development in ICAP‐1‐null mice is thymocyte‐autonomous.

### Role of ICAP‐1 in SP CD8^+^ thymocyte positive selection

**Figure 3:**
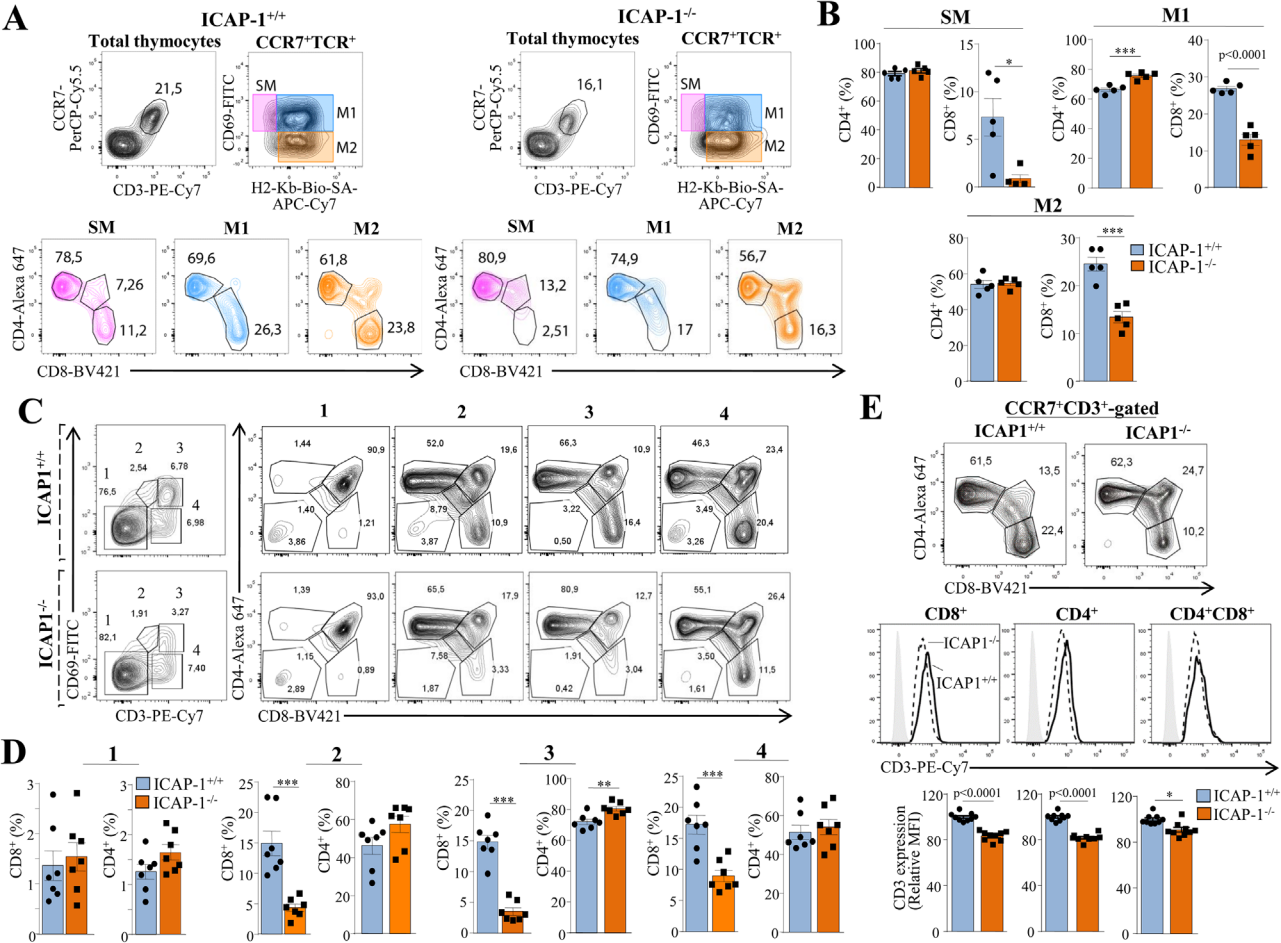
**Role of ICAP‐1 during SP CD8^+^ thymocyte positive selection**. Analysis of the multistep maturation stages encompassing positive selection. Cells were first gated on CCR7^+^CD3^+^, and the SM, M1 and M2 subpopulations were defined according to CD69 and H2‐Kb expression. Each of these populations was subsequently analyzed for the CD4 and CD8 levels. A representative dot plot result is shown (A), and data quantification of CD4^+^ and CD8^+^ proportions in the SM, M1 and M2 subsets is displayed in (B). (C) Cells were gated on CD3 and CD69 to mark the fractions 1 to 4, and each fraction was analyzed for CD4 and CD8 expression. Representative dot plots are shown, and panel (D) displays quantification of the data of CD4^+^ and CD8^+^ frequencies in fractions 1 to 4. (E) Analysis of CD3 expression on SP CD4^+^ and CD8^+^ thymocytes from ICAP‐1‐null and control mice was assessed by flow cytometry. Panels B, D, and E (bottom) show pooled data from two to four independent experiments (^***^ *p* < 0.001; ^**^ *p* < 0.01; ^*^ *p* < 0.05).

To dissect the alterations causing the deficient SP CD8^+^ cell generation in ICAP‐1^–/–^ mice, we first analyzed the expression of markers defining the multistep positive selection process [26, 27]. As TCR signals mediating positive selection lead to upregulated expression of the chemokine receptor CCR7 in DP thymocytes [28, 29, 30], we first gated on CCR7^+^TCR^+^ positively selected thymocytes, and subsequently analyzed coexpression of CD69 and MHC class I molecules, as described in Ref. [27]. We found a slight increase in H2‐Kb expression in CCR7^+^TCR^+^ thymocytes from ICAP‐1‐deficient mice, but it did not reach a statistical significance (not shown). CD69 and MHC class I expression was used to identify three distinct subsets of positively selected thymocytes at different maturation stages [27]. These subsets include (i) CD69^+^MHCI^–^ semimature (SM) cells which undergo apoptosis upon TCR engagement, (ii) CD69^+^MHCI^+^ mature 1 (M1) cells that are competent to proliferate, and (iii) CD69^–^MHCI^+^ mature 2 (M2) proliferating thymocytes, which express surface molecules allowing egress from thymus [26]. We next determined the proportions of SP CD4^+^ and CD8^+^ cells in the SM, M1, and M2 subsets (Fig. 3A), and quantification of the data revealed remarkable reductions of SP CD8^+^ thymocyte frequencies in the SM, M1, and M2 subpopulations in ICAP‐1‐null mice, while no alterations were observed in the SP CD4^+^ subsets (Fig. 3B), in line with a possible defective positive selection of SP CD8^+^ thymocytes.

We further studied the positive selection process by analyzing the expression of CD69 and CD3 on thymocytes, as reported in Ref. [31]. In this analysis, fraction 1 corresponds to preselection DP thymocytes; fraction 2 represents a transitional population after TCR engagement; fraction 3 shows the CD69^hi^CD3^hi^ post‐positive selected cells, and fraction 4 displays the more mature thymocyte population. We observed a reduced frequency of the fraction 3 cells in ICAP‐1‐deficient thymocytes (Fig. 3C, left). Of note, there was a marked decrease in SP CD8^+^ cell proportions in fractions 2, 3, and 4 from ICAP‐1^–/–^ mice compared to control counterparts, whereas minor increases in SP CD4^+^ percentages were detected (Fig. 3C and D). Together, these results reveal that impaired maturation of SP CD8^+^ thymocytes in ICAP‐1‐null mice is associated with alterations in the expression of developmental markers of positive selection. CD3 expression showed minor decreases on gated SP CD4^+^ and CD8^+^ (12–16%) and on DP (<9%) thymocytes from ICAP‐1‐null mice relative to their corresponding cell subsets in control mice (Fig. 3E).

### Unaltered α4β1‐dependent adhesion of ICAP‐1^–/–^ thymocytes

**Figure 4:**
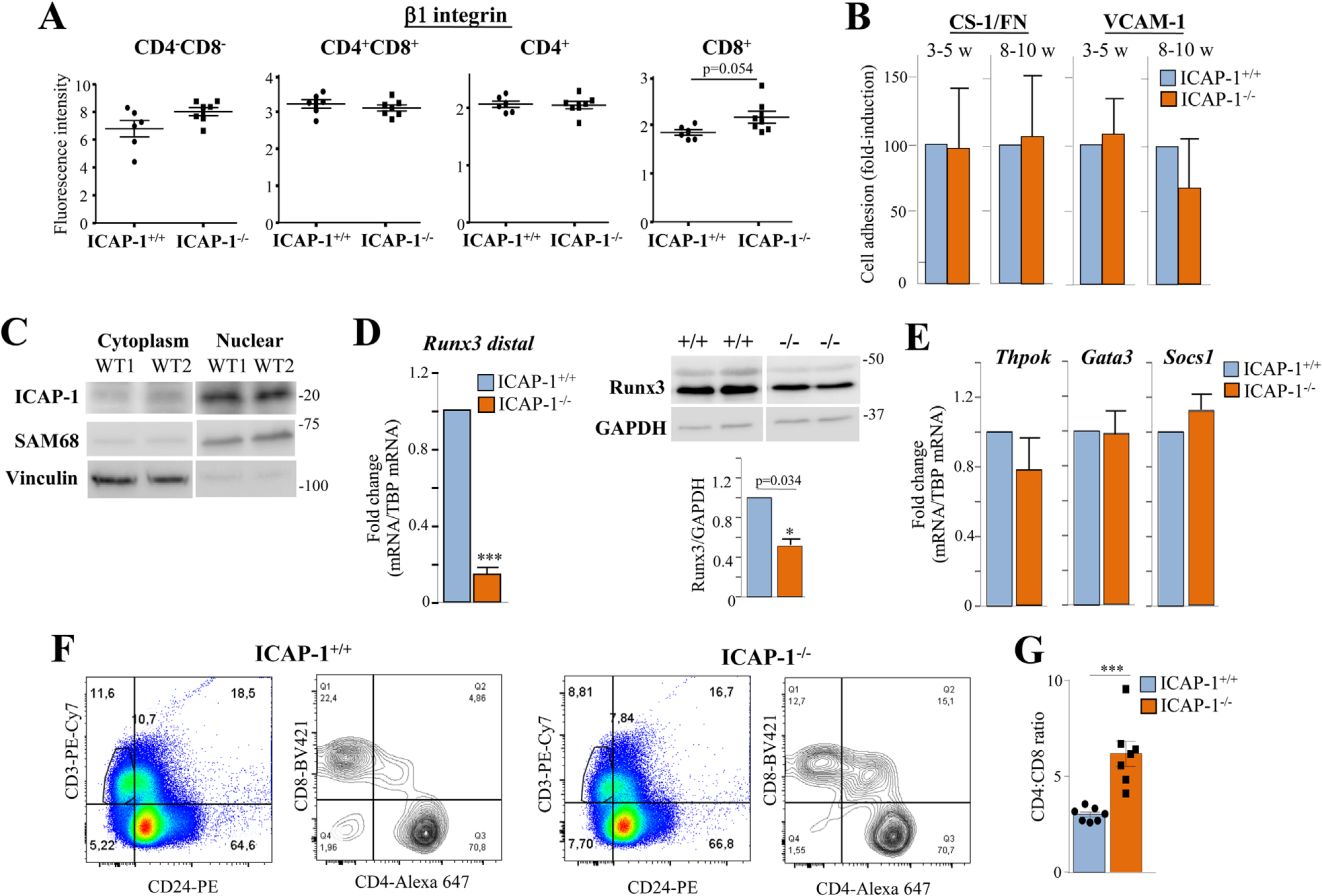
**Unaltered adhesive properties and reduced Runx3 levels in thymocytes from ICAP‐1^–/–^ mice**. (A) β1‐integrin expression on the indicated thymocyte subpopulations of 3‐ to 5‐week‐old control and ICAP‐1‐null mice was analyzed by flow cytometry. Data display pooled data from three independent experiments. (B) Thymocytes were subjected to adhesion assays to CS‐1/FN (n = 8) or to VCAM‐1 (n = 5). (C) Thymic cells from 3‐ to 5‐week‐old WT mice were subjected to cell fractionation assays to enrich for cytoplasmic and nuclear fractions. SAM68 and vinculin were used as nuclear and cytoplasmic markers, respectively. Shown is a representative result out of three independent experiments. (D, left) Expression of *Runx3*d in isolated SP CD8^+^ cells was determined by RT‐PCR (n = 3). (Right) Thymic cells were tested by immunoblotting to assess Runx3 expression (n = 3). (E) Thymic cells were tested by qPCR for expression of the indicated transcription factors (n = 3–4). (F) CD3^+^CD24^–^‐gated thymocytes were analyzed for CD4 and CD8 expression, and (G) the proportion of CD8^+^ cells coexpressing CD4 from these analyses is shown as CD4:CD8 ratios. A representative dot plot result is shown in (F), and panel (G) displays pooled data from three independent experiments (^***^ *p* < 0.001).

Lack of ICAP‐1 did not remarkably affect β1‐surface levels in different thymocyte subpopulations (Fig. 4A; Supporting information Fig. S4), and *Krit1* mRNA was barely detectable in thymus of control and ICAP‐1^–/–^ mice (not shown). The α4β1 integrin mediates thymocyte adhesion to the thymic epithelium [32, 33], although it has been demonstrated to be dispensable for thymic T‐cell differentiation [34, 35, 36]. Adhesion assays using ICAP‐1^+/+^ and ICAP‐1‐deficient thymocytes (85–90% DP cells) revealed similar adhesion levels to CS‐1/FN and VCAM‐1, regardless of adhesion times (2–4 min, Fig. 4B; 20–30 min, not shown), suggesting that altered SP thymocyte development in ICAP‐1‐null mice occurs under normal α4β1‐dependent thymocyte attachment.

### Reduced Runx3 levels in ICAP‐1‐deficient CD8^+^ SP thymocytes

In addition to binding β1 at the cell membrane, ICAP‐1 bears a nuclear localization signal which mediates its export to the nucleus [11, 12, 13]. Remarkably, ICAP‐1 displayed a strong nuclear distribution in thymic cells (Fig. 4C). As *Runx3* is a transcription factor required for SP CD8^+^ thymocyte generation [37, 38, 39, 40], we assessed its expression in isolated SP CD8^+^ cells. *Runx3* transcription is regulated by two different promoter regions, P1 (also called distal) and P2 [41]. The *Runx3* exon 1 is transcribed from the distal promoter, which is exclusively expressed in SP CD8^+^ cells [39]. Of note, RT‐PCR analyses using specific oligonucleotides to detect the *Runx3* distal (*Runx3*d) form revealed that *Runx3*d expression was markedly reduced in ICAP‐1‐null SP CD8^+^ cells (Fig. 4D, left). Furthermore, we found decreased Runx3 protein levels in thymus cells (Fig. 4D, right). In contrast, expression of *Thpok*, *Gata3*, and *Socs1*, which are transcription factors controlling SP CD4^+^ thymocyte production [42, 43, 44] was comparable between ICAP‐1^–/–^ and control cells (Fig. 4E).

Previous studies revealed that the development of DP thymocytes into SP CD4^+^ and CD8^+^ cells correlated with the loss of CD24 expression [45]. Furthermore, it has been demonstrated that Runx3‐deficiency leads to CD4 expression in thymic mature SP CD8^+^ T cells [40]. Interestingly, we found that decreased CD8^+^ SP cell frequency in selected CD3^+^CD24^–^ subset from ICAP‐1‐null mice was associated with a threefold increase in the proportion of CD8^+^ cells coexpressing CD4 (Fig. 4F and G). Together, these results raise the possibility that *Runx3* reduction might represent one of the mechanisms underlying the defective SP CD8^+^ cell development in ICAP‐1^–/–^ mice.

### Distribution and function of peripheral T cells in ICAP‐1‐null mice

**Figure 5:**
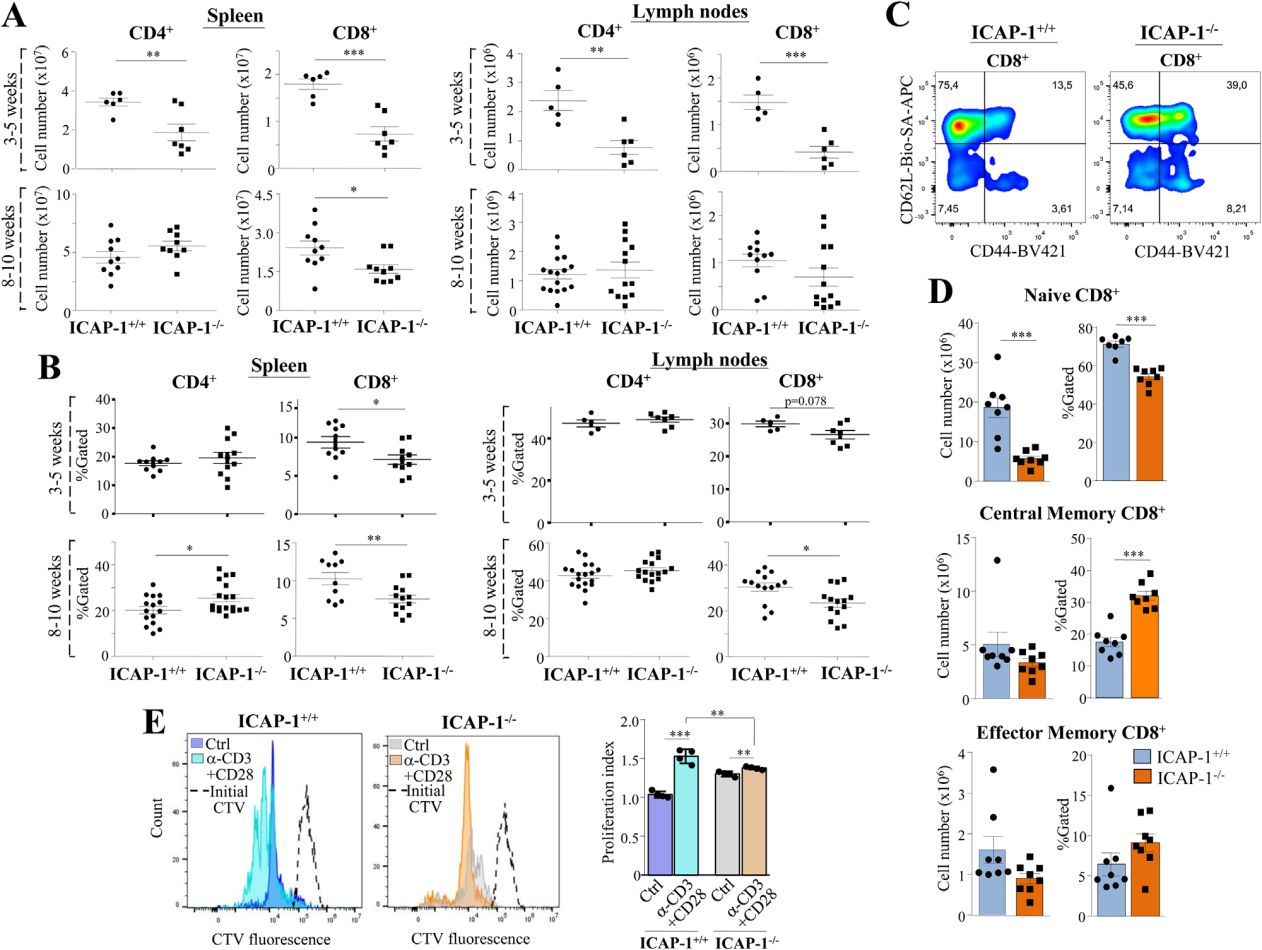
**Analysis of peripheral T‐cell distribution and function in ICAP‐1‐null mice**. Cell numbers (A) and frequencies (B) of CD4^+^ and CD8^+^ cells in spleens and LNs from ICAP‐1^+/+^ and ICAP‐1^–/–^ mice. (C, D) Proportions of naïve and memory CD8^+^ cells in control and ICAP‐1^–/‐^ spleens were assessed by FACS using the CD62L and CD44 markers. A representative dot plot result is shown on (C), and quantification of data (D) displays pooled data from three independent experiments. (E) CD3^+^‐selected spleen T cells were labeled with CellTracer Violet and incubated in the absence (Ctrl) or presence of coimmobilized anti‐CD3 and anti‐CD28 antibodies. Cell division (left) and the associated proliferation indexes (right) were analyzed by flow cytometry. Shown are representative dot plots and pooled data from two independent experiments. (^***^ *p* < 0.001; ^**^ *p* < 0.01, ^*^ *p* < 0.05).

Coincident with their smaller size, spleens from 3‐ to 5‐week old ICAP‐1^–/–^ mice displayed decreased total cellularity, whereas cell numbers were similar in spleens from 8‐ to 10‐week‐old animals (Supporting information Fig. S5A and B). The CD8^+^ cell numbers and proportions were lower in ICAP‐1‐null spleens and LNs than those in control mice (Fig. 5A and B; Supporting information Fig. S5C and D). CD4^+^ cell numbers displayed a reduction only in 3‐ to 5‐week old ICAP‐1‐deficient animals but not in older counterparts, but CD4^+^ cell proportions did not change or moderately increased (Fig. 5A and B; Supporting information Fig. S5C and D).

To look for potential alterations in naïve versus memory CD8^+^ cell distribution in ICAP‐1^–/–^ spleens, we analyzed the expression of CD62L and CD44 markers. Interestingly, numbers and proportions of naïve CD62L^hi^CD44^–^ cells were significantly reduced in ICAP‐1‐null CD8^+^ splenocytes (Fig. 5C and D). The total numbers of CD8^+^ central memory CD62L^hi^CD44^+^ and effector memory CD62L^–^CD44^hi^ cells were not remarkably altered in the mutant animals, but their proportions increased, especially in central memory CD8^+^ cells (Fig. 5C and D).

As an assessment of the function of peripheral T cells lacking ICAP‐1, we subjected spleen T cells to in vitro proliferation assays. CD3^+^‐selected, CellTracer Violet (CTV)‐labelled T splenocytes were incubated with or without plate‐bound anti‐CD3 and anti‐CD28 antibodies, and the extent of proliferation assessed by FACS. Of note, CD3^+^‐gated spleen T cells from ICAP‐1^–/–^ mice displayed significantly less division and proliferation rates than WT counterparts in response to the stimuli (Fig. 5E; Supporting information Fig. S5E).

### Distribution and function peripheral B cells in ICAP‐1‐null mice

**Figure 6:**
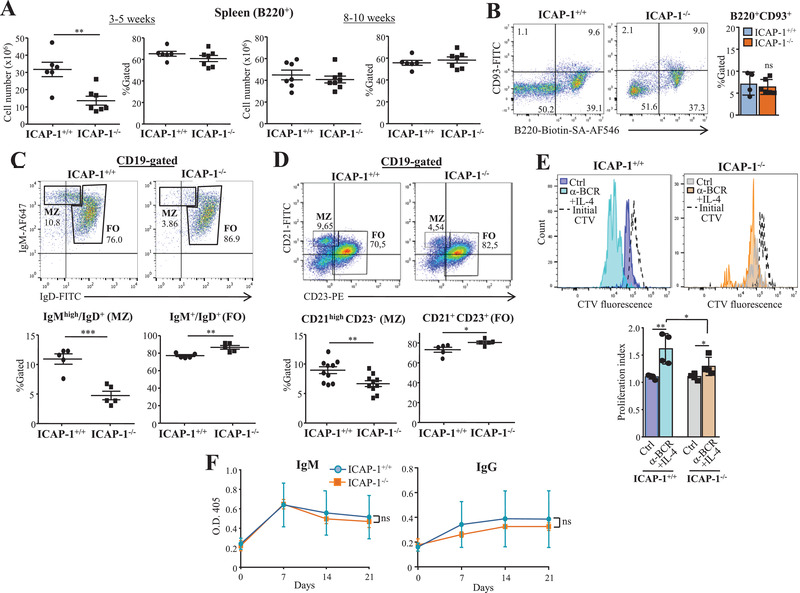
**Altered marginal zone (MZ) and follicular (FO) B‐cell proportions, and reduced B‐cell proliferation in spleens from ICAP‐1‐null mice**. (A) Number and proportions of B220^+^ cells in spleens from ICAP‐1^+/+^ and ICAP‐1^–/–^ mice were assessed by flow cytometry. (B, left) Representative dot plots showing the CD93^+^ immature B‐cell fraction in spleen B220^+^ cells from 8‐ to 10‐week‐old mice. (Right) Data show pooled data from two independent experiments. (C, D) The proportions of MZ and FO CD19^+^‐gated spleen B cells were determined by FACS with anti‐IgM or anti‐IgD (C), or with anti‐CD21 and anti‐CD23 antibodies (D). Top panels show representative dot plots, and bottom results display pooled data from two to four independent experiments. (E) CD19^+^‐selected spleen B cells were labeled with CellTracer Violet and incubated in the absence (Ctrl) or presence of anti‐BCR and IL‐4. Cell division and the associated proliferation indexes were analyzed by flow cytometry. Shown is a representative result out of two independent experiments. (F) Analysis by ELISA of TNP‐specific IgM and IgG antibodies at different days postimmunization in the serum of control and ICAP‐1‐deficient mice. Shown is a representative result out of two independent experiments (^***^ *p* < 0.001, ^**^ *p* < 0.01, and ^*^ *p* < 0.05).

Reflecting the reduced cell numbers in the spleen (see Supporting information Fig. S5B), we found decreased B220^+^ cellularity only in spleens from 3‐ to 5‐week‐old ICAP‐1‐null mice, but B220^+^ cell proportions were unaltered in these mice as well as in older animals (Fig. 6A; Supporting information Fig. S6A). Analysis of the B220^+^CD93^+^ immature B‐cell proportions in spleens revealed no remarkable alterations in ICAP‐1^–/–^ mice relative to control counterparts (Fig. 6B). In addition, B220^+^ cell numbers and frequencies were similar in LNs from control and ICAP‐1‐deficient mice (Supporting information Fig. S6B).

B cells in the spleen undergo transitional stages into mature follicular (FO) IgM^+^IgD^+^ cells or marginal zone (MZ) IgM^high^/IgD^+^ B cells [46, 47]. Notably, the proportion of CD19‐PE‐gated spleen B cells displaying a MZ‐like phenotype was markedly lower in ICAP‐1‐null mice than control littermates, whereas a moderate increase in FO cell frequencies in ICAP‐1^–/–^ mice was observed (Fig. 6C). We further evaluated MZ and FO B cells based on CD21 and CD23 expression which revealed reduced CD21^high^CD23^–^ MZ and enhanced CD21^+^CD23^+^ FO cell proportions in CD19‐APC‐gated cells from ICAP‐1^–/–^ mice (Fig. 6D, Supporting information Fig. S6C). Immunohistology using anti‐IgM and anti‐IgD antibodies showed similar B‐cell distributions in the white pulps of control and ICAP‐1^–/–^ mice, and evaluation of the MZ showed no differences in thickness compared to control animals (Supporting information Fig. S6D). Furthermore, the structure of follicles and paracortex appeared normal in ICAP‐1^–/–^ mice, and we observed no significant alterations in the formation of B‐cell follicles or the MZ located near MOMA‐1^+^ metallophilic macrophages (Supporting information Fig. S6D). The discrepancy between the flow cytometry and histology data on the MZ analyses could be related to the differences in sample processing. While lower frequencies of MZ B cells are detected by flow cytometry in the homogenized tissue of ICAP‐1‐null mice, these cells could be able and enough in numbers to distribute within the whole MZ niche, and when analyzed by immunofluorescence of tissue sections, we might not detect clear differences between ICAP‐1^–/–^ and control mice in relation with thickness.

To examine the function of peripheral B cells lacking ICAP‐1, we first assessed in vitro B‐cell proliferation in the presence of anti‐BCR plus IL‐4. CTV‐labelled, CD19^+^‐selected spleen B cells from ICAP‐1‐deficient mice proliferated significantly less than control cells (Fig. 6E). We next compared T‐independent, B‐cell specific humoral immune responses by immunization with TNP‐Ficoll (a TI‐2 antigen), measuring by ELISA the levels of TNP‐specific antibodies in the serum at different days postimmunization. Data revealed no major differences in IgM and IgG serum content between control and ICAP‐1‐null mice (Fig. 6F). These results indicate that ICAP‐1 does not play significant roles in B‐cell antibody responses to T‐independent antigens.

### Adhesion properties of ICAP‐1‐deficient splenocytes

**Figure 7:**
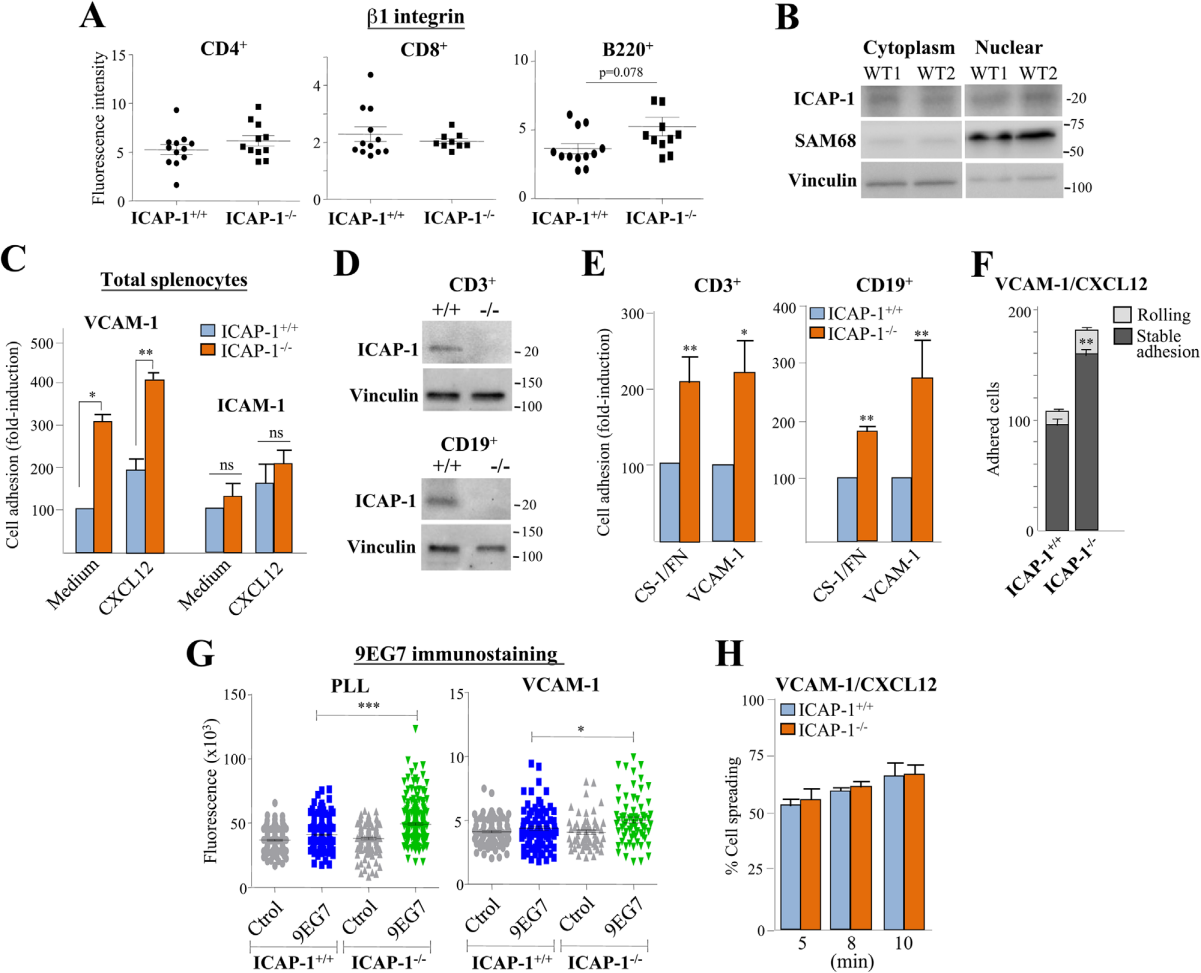
**ICAP‐1^–/–^ splenocytes display increased α4β1‐dependent attachment**. (A) β1‐ integrin expression on the indicated T‐ and B‐spleen cell populations was analyzed by FACS. Data show pooled data from four independent experiments. (B) Spleen cells from WT mice were subjected to cell fractionation assays as in Figure 4. Shown is a representative result out of three independent experiments. (C) Total splenocytes from ICAP‐1^+/+^ or ICAP‐1^–/–^ mice were subjected to static adhesion assays to VCAM‐1 or ICAM‐1 coimmobilized without (Medium) or with CXCL12 (n = 3‐6). CD3^+^‐ or CD19^+^‐selected T or B cells from ICAP‐1^+/+^ or ICAP‐1‐null spleens were analyzed by immunoblotting for ICAP‐1 expression (D), or tested in static adhesion assays to VCAM‐1 or CS‐1/FN (n = 3) (E). (F) Splenocytes were perfused in flow chambers coated with VCAM‐1 coimmobilized with CXCL12, and analyzed for rolling and stable cell arrest. Data are presented as mean ± SD of adhered cells (n = 3). (G) Splenocytes were attached onto PLL or VCAM‐1, incubated with isotype control or with the 9EG7 anti‐β1 mAb and subjected to fluorescence microscopy. Shown is a representative result out of two independent experiments. (H) Splenocytes were allowed to attach to VCAM‐1 coimmobilized with CXCL12, and spreading was evaluated from Nomarski images at the indicated times. Percentage of cell spreading was determined from cells (2500–3000) from different fields of view (n = 2) (^***^ *p* < 0.001, ^**^ *p* < 0.01, ^*^ *p* < 0.05).

β1‐integrin expression on spleen CD4^+^ and CD8^+^ cells of ICAP‐1^–/–^ and ICAP‐1^+/+^ mice was mostly comparable, whereas a tendency to increased expression was detected on B220^+^ cells (Fig. 7A; Supporting information Fig. S7A). Examination of β1‐levels separately on MZ and FO B cells, revealed only a minimal increase in expression in both cell subsets (Supporting information Fig. S7B). In addition, ICAP‐1 loss did not affect talin and kindlin‐3 expression (Supporting information Fig. S7C), and *KRIT1* mRNA remained almost undetectable in control and ICAP‐1‐deficient spleens (not shown). Unlike thymic cells, ICAP‐1 expression was more evenly distributed between cytoplasm and nuclear fractions of spleen cells (Fig. 7B). Notably, ICAP‐1‐null total splenocytes displayed stronger adhesion to α4β1 ligands than control cells, and in support of lack of ICAP‐1 binding to β2‐integrins [6, 7], there were no changes in adhesion to ICAM‐1 between control and ICAP‐1^–/–^ splenocytes (Fig. 7C). Moreover, spleen‐derived selected CD3^+^ T and CD19^+^ B cells showed similar levels of ICAP‐1 expression and upregulation of α4β1‐dependent attachment (Fig. 7D and E). Results from adhesion assays under shear stress measuring splenocytes attached to VCAM‐1 coimmobilized with CXCL12 showed higher numbers of stably arrested ICAP‐1^–/–^ cells than control cells (Fig. 7F), confirming that ICAP‐1 negatively regulates α4β1‐mediated splenocyte adhesion. The 9EG7 mAb, which binds to an activation‐induced epitope on murine β1‐integrins [48], displayed stronger binding to ICAP‐1^–/–^ splenocytes than to control counterparts (Fig. 7G), indicating that ICAP‐1 controls the β1‐integrin affinity of splenocytes. Finally, ICAP‐1^+/+^ and ICAP‐1^–/–^ splenocytes exhibited similar spreading on VCAM‐1/CXCL12 (Fig. 7H), suggesting that ICAP‐1 does not regulate α4β1‐mediated postligand binding events.

## Discussion

Using ICAP‐1‐null mice, we have investigated the role of ICAP‐1 in the regulation of immune cell differentiation and function. We found that the HSC and HPC compartments in the BM as well as B lymphopoiesis were not remarkably affected by ICAP‐1 loss. Examination of the development of the different thymocyte subsets revealed a defective generation of mature SP TCRαβ^+^CD8^+^ thymocytes in mice lacking ICAP‐1, thus, unveiling an ICAP‐1 involvement in SP thymocyte development. Based on the marked reductions in the proportions of the SM, M1 and M2 SP CD8^+^ cellular subsets [26], as well as on the decreased frequencies of SP CD8^+^ thymocytes within the CD69^+^CD3^+^ and the more mature CD69^–^CD3^+^ subsets in ICAP‐1‐null mice, we concluded that lack of ICAP‐1 causes a defective maturation of SP CD8^+^ cell. The altered expression of different markers associated with positive selection suggests an impairment of this process in the SP CD8^+^ thymocyte lineage in ICAP‐1‐deficient mice, but we cannot formally exclude that ICAP‐1 could also affect the negative selection process.

Adhesion assays to α4β1 ligands using total thymocytes (mostly DP) showed similar attachment levels between ICAP‐1^–/–^ and control thymocytes, suggesting that defective SP thymocyte development in ICAP‐1‐null mice is probably independent of potential adhesion alterations. We have not determined if ICAP‐1 absence in selected SP CD4^+^ and CD8^+^ thymocytes affects their α4β1‐dependent attachment. ICAP‐1 bears a nuclear localization signal that has been shown to facilitate ICAP‐1 shuttling into the nucleus of fibroblasts and epithelial cells, with functional consequences on transcriptional activities [11, 12, 13]. Interestingly, ICAP‐1 showed a strong preferential localization in the nucleus of thymic cells, and it is therefore, tempting to hypothesize that the ICAP‐1 predominant nuclear localization might be responsible for its lack of regulation of α4β1‐mediated thymocyte adhesion.

Interactions of TCRs on DP thymocytes with self‐peptides presented by MHC molecules on TECs regulate the expression of transcription factors that ultimately direct cell fates and positive selection toward the CD4^+^ or CD8^+^ lineages [43, 49, 50]. The transcription factor Runx3 is required for SP CD8^+^ cell development from thymic DP cells and for repression of CD4 expression [37‐39, 43]. Intriguingly, we have detected a direct correlation between the lack of ICAP‐1 and strongly reduced *Runx3* mRNA levels in isolated SP CD8^+^ thymocytes. Moreover, we found an increment in the frequency of CD8^+^CD3^+^CD24^–^ mature thymocytes that coexpress CD4 in ICAP‐1‐null mice, a result in line with the observed CD4 expression in mature SP CD8^+^ T cells in Runx3‐deficient mice [40]. These data raise the possibility of a potential involvement of nuclear ICAP1 in the regulation of *Runx3* expression, which could be behind the deficient transcriptional regulation of CD8 that results in the altered development of SP CD8^+^ thymocytes in ICAP‐1^–/–^ mice. Notably, decreased expression of the bone marker *Runx2*, another member of the RUNX transcription family [51], was also observed in ICAP‐1‐null mice [18]. As selected thymocytes contribute to the development of mTECs [52, 53], it remains an open question whether the impaired production of mature SP CD8^+^ thymocytes in ICAP‐1‐deficient mice impacts the maturation of their mTECs.

The strength and duration of signaling transduced by TCR is also important for controlling the development of CD4^+^ and CD8^+^ thymocytes, so that strong and durable signaling are associated with CD4^+^ development, whereas weak and short signaling is linked to the generation of CD8^+^ thymocytes (reviewed in [43]). A key kinase needed in early TCR signaling is ZAP‐70, whose increased abundance is required in DP3 thymocytes for the efficient generation of SP CD8^+^ cells [54]. Therefore, it will be interesting to characterize the TCR‐dependent signaling players in ICAP‐1‐null mice, including ZAP‐70, to assess their potential links to the observed defective SP CD8^+^ thymocyte development.

Reduced cell numbers and proportions of spleen CD8^+^ T cells in ICAP‐1^–/–^ mice were exclusively found in the naïve CD62L^hi^CD44^–^ subset, which could likely reflect the reduced CD8^+^ cell production from their thymi. The decrease in CD8^+^ cell numbers in LNs from ICAP‐1‐null mice could also correlate with the diminished SP CD8^+^ thymic cell production, but it could also be accounted for by the reduced proliferation of T cells, as we found that ICAP‐1^–/–^ T cells have lower proliferation rates in response to combined CD3 plus CD28 antibodies. Interestingly, reduced proliferation of T‐cell splenocytes in response to anti‐CD3 plus anti‐CD28 was described in Runx3^–/–^ mice [37], again revealing functional similarities with ICAP‐1‐deficient mice.

Both total and CD3^+^‐selected splenocytes from ICAP‐1^–/–^ mice showed an upregulation of adhesion to α4β1 ligands, indicating that ICAP‐1 negatively controls their α4β1‐dependent attachment. Thus, the more even distribution of ICAP‐1 in the cytoplasm and nucleus of splenocytes might contribute to ICAP‐1 regulation of their α4β1‐mediated adhesion, as compared to the predominant nuclear localization of ICAP‐1 in thymocytes. The higher α4β1‐dependent adhesion of ICAP‐1^–/–^ spleen T cells could open the likelihood that an increased number of T cells might be retained in the splenic T‐cell zone. However, as mentioned above, this does not seem the case for CD8^+^ cells, as their diminished frequencies in the spleens and LNs of ICAP‐1^–/–^ mice most likely reflect the reduced CD8^+^ cell production from their thymi. Nonetheless, we cannot exclude intrinsic alterations in trafficking to and within these lymphoid organs, as we detected increased PB CD8^+^ cells in ICAP‐1^–/–^ mice linked to the reduced CD8^+^ cell numbers in spleen and LNs.

Correlating with the observed in vitro spleen B cell upregulated attachment to α4β1 ligands, a remarkable decrease in MZ B‐cell frequencies and a moderate increase in FO B cells was observed in ICAP‐1‐null spleens, without gross changes in the splenic microarchitecture. Retention of MZ B cells in the MZ depends on α4β1 and αLβ2, whereas these integrins are not required for FO B cell lodging in the follicles [55]. The frequency of mature B cells in the BM was similar between ICAP‐1^–/–^ and control mice, indicating that enhanced FO B‐cell percentages in ICAP‐1‐deficient spleens do not lead to their increased recirculation to the BM. An enhanced α4β1‐dependent MZ B‐ cell adhesion could theoretically augment their retention in the MZ, causing a reduction in their shuttling to the follicles, although total cell numbers should not change. We have not tested if reduced MZ B‐cell frequency is associated with potential MZ B‐cell egress from the spleen. Regulated cell trafficking in the spleen is one of the several mechanisms controlling MZ B‐cell maturation [46]. These mechanisms are dictated by commitment signals to a MZ B‐cell fate such as those depending on the strength of BCR signaling, Notch2 activity, BAFF‐BAFFR interactions and the NF‐κB pathway [46, 56‐59]. Whether any of such processes is perturbed in ICAP‐1^–/–^ spleens dependently or independently of the altered α4β1‐mediated B‐cell adhesion, whether increased apoptosis of ICAP‐1‐deficient B cells occurs, or alternatively, if inhibition of MZ B‐cell generation from their precursors takes place, warrants further studies.

Finally, reduced proliferation in response to soluble, adhesion‐independent anti‐BCR plus IL‐4 stimuli was found in CD19^+^ B cells from ICAP‐1‐null mice. We have not yet addressed whether early B‐cell activation events are altered in ICAP‐1^–/–^ spleen B cells, or if the deficient proliferation might be linked with defective cell survival. The fact that ICAP‐1‐deficient spleen T cells also display reduced proliferation in response to stimuli provided by CD3 and CD28 antibodies, suggests that ICAP‐1 can regulate lymphocyte proliferation. It will be important to unveil the molecular mechanisms associated with this regulation.

Collectively, the results from this study unravel an involvement of ICAP‐1 in the generation and/or maintenance of CD8^+^ SP thymocytes and MZ B cells, and indicate that the distinct ICAP‐1 functions might be controlled by its cellular localization.

## Materials and methods

### Mice and cells

ICAP^+/–^ mice were intercrossed to generate ICAP^–/–^ mice that were genotyped by PCR [18]. Confirmation of ICAP‐1 deletion was further assessed by immunoblotting with anti‐ICAP‐1 antibodies [18]. Mice were kept under pathogen‐free conditions at the Animal Facility of the Centro de Investigaciones Biológicas. Single‐cell suspensions from spleens, LNs, thymus, and BM from femur and tibia were obtained from 3‐ to 5‐ or 8‐ to 10‐week‐old male or female mice. Spleen CD3^+^ T cells were enriched by negative selection (EasySep, Stem Cell Technologies, Vancouver, Canada) (purity > 85%), or were positively selected by cell sorting (FACSAria Fusion, Becton‐Dickinson) using an Alexa Fluor647 anti‐mouse CD3ε antibody (see Supporting information Table S1). SP CD8^+^ thymocytes were isolated with the MagCellect Mouse CD8^+^ T‐Cell Isolation Kit (R&D Systems, Minneapolis, MN; purity > 80%). DP CD4^+^CD8^+^ and SP CD4^+^ thymocytes were stained with FITC‐anti‐mouse CD4^+^ and PE‐anti‐mouse CD8^+^ antibodies (Supporting information Table S1), and positively selected by cell sorting. CD19^+^ spleen B cells were isolated (purity > 90%) by cell depletion with the MagCellect Mouse B‐Cell Isolation Kit (R&D Systems, purity > 90%). TECs enrichment was achieved following the described method [60]. For the preparation of chimeric mice, BM cell suspensions from donors (6‐ to 7‐week‐old) were intravenously injected into lethally irradiated (twice 600 rad doses, separated by 3 h) recipient animals. Thymocyte populations from recipient mice were analyzed by flow cytometry 6–7 weeks posttransplantation. All T‐cell assays comply with the guidance provided by MIATA.

### Flow cytometry

Cells were stained on ice with fluorochrome‐conjugated antibodies, followed by incubation with secondary antibodies or streptavidin‐conjugated fluorochromes. Conjugated Rat IgG2b κ and Armenian Hamster IgG were used as isotype controls. The antibodies used for FACS are listed in Supporting information Table S1. For characterization of TECs, cells were incubated with UEA1‐Biotin (Vector Laboratories, Burlingame, CA), which was detected by incubating with streptavidin‐APC (BioLegend, San Diego, CA). Samples were analyzed either with a FC500 or a Cytoflex‐S (Beckman Coulter), with a FACs Canto II, or with a FACS Calibur (BD Biosciences). We used CXP, FlowLogic, FlowJo X (Tree Star, Inc), FlowJo v10.7.1, or FCS Express III software for analysis of FACS data. We have adhered to the guidelines for the use of flow cytometry and cell sorting in immunological studies (https://onlinelibrary.wiley.com/doi/full/10.1002/eji.202170126).

### Cell adhesion and spreading assays

Static adhesion assays to α4β1 ligands were carried out as described [61]. In brief, labeled cells were plated on wells coated with the fibronectin fragment FN‐H89 (CS‐1/FN) or with recombinant VCAM‐1 or ICAM‐1 (R&D Systems), in the absence or presence of immobilized CXCL12 (R&D Systems). Plates were incubated for 2–4 min at 37°C after a short spin and adhesions quantified with a fluorescence analyzer. Adhesion data are presented relative to control cells, which were given an arbitrary value of 100. For flow chamber adhesion assays [61], cells were infused at 1 dyn/cm^2^ into flow chambers containing coimmobilized VCAM‐1 and CXCL12. Rolling cells subsequently attached were expressed as stable arrest, whereas tethered cells not arrested were expressed as rolling cells. For cellular spreading, CXCL12‐stimulated cells attached on VCAM‐1 were analyzed as previously described [61].

### Analysis of high‐affinity β1‐integrins

Splenocytes were cultured for 1 h at 37°C onto slides (Ibidi GmbH, Planegg, Germany) coated with poly‐l‐lysine (PLL; Sigma Aldrich, St. Louis, MO) or with Protein A (Sigma Aldrich)/mouse VCAM‐Fc (R&D), and subsequently fixed with 4% paraformaldehyde in PBS. Then cells were blocked with Fc receptor‐blocking antibodies (rat anti‐mouse CD16/CD32, BD Biosciences), followed by incubation at 37°C with the 9EG7 rat anti‐mouse β1 mAb (BD Biosciences) or with isotype controls, and finally incubated with fluorochrome‐conjugated secondary antibodies. Samples were mounted with ProLong diamond antifade mountant with DAPI (Molecular Probes, Eugene, OR), and were analyzed using a fluorescence microscope CCD Digital Camera Leica DFC 350 FX, coupled with a light microscopy Zeiss Axioplan with a 100× oil immersion objective.

### Immunoblotting and cell fractionation

For immunoblotting, cells were solubilized as described [62]. After SDS‐PAGE, proteins were transferred to PVDF membranes and incubated with primary antibodies and HRP‐conjugated secondary antibodies (Jackson ImmunoResearch). Proteins were visualized using Immobilon Western (Millipore, Billerica, MA) and a chemiluminiscence detector (Fujifilm LAS 3000 Image Reader). For cell fractionation, cells were lysed in ice (HEPES 10 mM pH 8.0, KCl 10 mM, MgCl_2_ 1.5 mM, sucrose 34 mM, glycerol 10%, DTT 1 mM, and Triton X‐100 0.1%, supplemented with protease inhibitors; CFL buffer), and subsequently samples were centrifuged at 3500 rpm for 5 min at 4°C. Supernatant represented the cytosolic fraction. The pellet was washed once with CFL buffer, sonicated in lysis buffer, followed by centrifugation at high speed for 15 min at 4°C. The resulting supernatant was considered the nuclear fraction, and proteins from each pool were tested by immunoblotting according to the described method [62]).

### In vitro cell proliferation and T‐independent immunization assays

For T‐cell proliferation assays, purified spleen CD3^+^ cells from 8‐ to 10‐week old mice were labelled with 5 μM CTV (Thermo Fisher, Waltham, MA). After washing, cells were resuspended in RPMI 1640 medium supplemented with 10% fetal bovine serum, 2 mM l‐glutamine, 50 μM β‐mercaptoethanol, and nonessential amino acids (400 μM). CTV‐labelled cells (2 × 10^5^/well; counted using a hemocytometer) were cultured at 37°C in p96 flat‐bottom plates coated without (PBS) or with anti‐CD3 (clone 145‐2C11, BioLegend) plus anti‐CD28 (clone 37.51, BioLegend). Cells were harvested at 72 h, CD3‐labelled for T‐cell identification, and analyzed by flow cytometry. Proliferation indexes were obtained with FlowJo analysis software (BD Biosciences), showing the ratio of the total number of divisions between the number of cells that went into division. For B‐cell proliferation assays, we followed the described method [63]. Briefly, CTV‐labelled CD19^+^ B cells were cultured with goat anti‐BCR (20 μg/mL; Jackson Immunoresearch, Ely, UK) plus recombinant mouse IL‐4 (20 ng/mL Peprotech, London, UK), and after 96 h, cells were analyzed by flow cytometry and proliferation indexes obtained using FlowJo analysis software. For T‐independent immunization assays, groups of four control and ICAP‐1‐null mice were immunized with TNP‐Ficoll (Biosearch Technologies) as described in Ref, [63]. Peripheral blood samples were taken 24 h before immunization, and at days 7, 14, and 21 postimmunization. Serum levels of TNP‐specific IgM and IgG antibodies were evaluated in triplicates by ELISA. Antibodies bound to specific antigens were detected with biotinylated goat anti‐mouse IgM and anti‐IgG antibodies, respectively (Southern Biotech, Birmingham, AL), plus alkaline phosphatase‐streptavidin (SIGMA). After *p*‐nitrophenyl phosphate addition, plates were measured at 405 nm in a plate reader at 45 min after the reaction.

### Real‐time quantitative PCR

For mRNA expression analyses, RT‐qPCR was performed in triplicate using iQ SYBR Green Supermix (Bio‐Rad Laboratories) and LightCycler 480 II (Roche). Results were normalized according to the expression levels of *TBP* (TATA‐binding protein) RNA. Sequences for oligonucleotides are shown in Supporting Information Table S2.

### Statistical analyses

Analyses were performed with GraphPad Prism 5. Outliers according to the Grubbs' test were excluded. Two groups of normally distributed data were compared using the paired and unpaired *t*‐test. The results were considered significantly different when *p* < 0.05. Nonsignificant differences were marked as ns. Error bars show ±SD.

## Funding sources

This work was supported by grants SAF2017‐85146‐R and PID2020‐116291RB‐I00 from the Ministerio de Ciencia e Innovación (MICINN) to J.T., PID2019‐105623RB‐I00 from MICINN to M.L.T., BFU2013‐48828‐P from MICINN to Y.R.C., ERC Synergy Grant (2018) to R.F., RTI2018‐095497‐B‐I00 from MICINN to A.H, and RTI2018‐093938‐B‐I100 from MICINN, and (RD16/0011/0002, TERCEL) from Instituto de Salud Carlos III to A.G.Z.

## Conflict of interest

The authors declare no commercial or financial conflict of interest.

## Author contributions

JT wrote the manuscript. Experimental design, data analysis, and interpretation were performed by SSM, PF, PWK, SIV, JGC, DB, AGP, AGZ, AH, RF, YRC, MLT, and JT. Experimental performance was performed by SSM, PF, NAS, YRG, PWK, SIV, SMH, JGC, VBH, SRG, NAM, CBA, and GC.

## Ethics approval statement

All mouse experiments were approved by the Consejo Superior de Investigaciones Científicas Ethics Committee.

### Peer review

The peer review history for this article is available at https://publons.com/publon/10.1002/eji.202149560.

**Abbreviations**
CS‐1/FN: connecting segment 1/fibronectin
CTV: CellTracer Violet
DN: double negative
DP: double positive
FO: follicular
HSPC: hematopoietic stem and progenitor cell
ISP: immature single positive
MZ: marginal zone
PLL: poly‐l‐lysine
SP: single positive
TEC: thymic epithelial cell

## Supporting information

Fig. S1. Bone marrow and thymic properties of ICAP‐1‐null mice
Fig. S2. Analysis of thymocyte subpopulations in ICAP‐1‐deficient mice
Fig. S3. Characterization of thymic epithelial cells, thymus architecture, and analysis of BM chimerae
Fig. S4. Expression of b1 (CD29) in thymocyte subsets from ICAP‐1‐deficient mice
Fig. S5. Spleen and lymph node cell numbers, gating strategies and functional assessment of spleen T cells.
Fig. S6. Spleen and lymph node B cell distribution and B cell proliferation analyses
Fig. S7. Expression of b1, talin and kindlin‐3 in spleen cells from ICAP‐1‐deficient mice
Table S1. List of antibodies used for flow cytometry
Table S2. Oligonucleotide sequences used for qRT‐PCR assays

## Data Availability

The data that support the findings of this study are available from the corresponding author upon request.

## References

[1] Hynes, R. O. , Integrins: bidirectional, allosteric signaling machines. Cell 2002. 110: 673–687. 12297042 10.1016/s0092-8674(02)00971-6

[2] Moser, M. , Legate, K. R. , Zent, R. and Fassler, R. , The tail of integrins, talin, and kindlins. Science 2009. 324: 895–899. 19443776 10.1126/science.1163865

[3] Bouvard, D. , Pouwels, J. , De Franceschi, N. and Ivaska, J. , Integrin inactivators: balancing cellular functions in vitro and in vivo. Nat. Rev. Mol. Cell Biol. 2013. 14: 430–442. 23719537 10.1038/nrm3599

[4] Kim, C. , Ye, F. and Ginsberg, M. H. , Regulation of integrin activation. Annu. Rev. Cell Dev. Biol. 2011. 27: 321–345. 21663444 10.1146/annurev-cellbio-100109-104104

[5] Calderwood, D. A. , Campbell, I. D. and Critchley, D. R. , Talins and kindlins: partners in integrin‐mediated adhesion. Nat. Rev. Mol. Cell Biol. 2013. 14: 503–517. 23860236 10.1038/nrm3624 PMC4116690

[6] Chang, D. D. , Wong, C. , Smith, H. and Liu, J. , ICAP‐1, a novel beta1 integrin cytoplasmic domain‐associated protein, binds to a conserved and functionally important NPXY sequence motif of beta1 integrin. J. Cell Biol. 1997. 138: 1149–1157. 9281591 10.1083/jcb.138.5.1149 PMC2136751

[7] Zhang, X. A. and Hemler, M. E. , Interaction of the integrin beta1 cytoplasmic domain with ICAP‐1 protein. J. Biol. Chem. 1999. 274: 11–19. 9867804 10.1074/jbc.274.1.11

[8] Chang, D. D. , Hoang, B. Q. , Liu, J. and Springer, T. A. , Molecular basis for interaction between Icap1 alpha PTB domain and beta 1 integrin. J. Biol. Chem. 2002. 277: 8140–8145. 11741908 10.1074/jbc.M109031200

[9] Bouvard, D. , Vignoud, L. , Dupe‐Manet, S. , Abed, N. , Fournier, H. N. , Vincent‐Monegat, C. , Retta, S. F. et al., Disruption of focal adhesions by integrin cytoplasmic domain‐associated protein‐1 alpha. J. Biol. Chem. 2003. 278: 6567–6574. 12473654 10.1074/jbc.M211258200

[10] Brunner, M. , Millon‐Fremillon, A. , Chevalier, G. , Nakchbandi, I. A. , Mosher, D. , Block, M. R. , Albiges‐Rizo, C. and Bouvard, D. , Osteoblast mineralization requires beta1 integrin/ICAP‐1‐dependent fibronectin deposition. J. Cell. Biol. 2011. 194: 307–322. 21768292 10.1083/jcb.201007108 PMC3144405

[11] Fournier, H. N. , Dupe‐Manet, S. , Bouvard, D. , Luton, F. , Degani, S. , Block, M. R. , Retta, S. F. and Albiges‐Rizo, C. , Nuclear translocation of integrin cytoplasmic domain‐associated protein 1 stimulates cellular proliferation. Mol. Biol. Cell 2005. 16: 1859–1871. 15703214 10.1091/mbc.E04-08-0744 PMC1073667

[12] Draheim, K. M. , Huet‐Calderwood, C. , Simon, B. and Calderwood, D. A. , Nuclear localization of integrin cytoplasmic domain‐associated protein‐1 (ICAP1) influences beta1 integrin activation and recruits Krev/Interaction trapped‐1 (KRIT1) to the nucleus. J. Biol. Chem. 2017. 292: 1884–1898. 28003363 10.1074/jbc.M116.762393 PMC5290960

[13] Su, V. L. , Simon, B. , Draheim, K. M. and Calderwood, D. A. , Serine phosphorylation of the small phosphoprotein ICAP1 inhibits its nuclear accumulation. J. Biol. Chem. 2020. 295: 3269–3284. 32005669 10.1074/jbc.RA119.009794 PMC7062153

[14] Millon‐Fremillon, A. , Brunner, M. , Abed, N. , Collomb, E. , Ribba, A. S. , Block, M. R. , Albiges‐Rizo, C. and Bouvard, D. , Calcium and calmodulin‐dependent serine/threonine protein kinase type II (CaMKII)‐mediated intramolecular opening of integrin cytoplasmic domain‐associated protein‐1 (ICAP‐1alpha) negatively regulates beta1 integrins. J. Biol. Chem. 2013. 288: 20248–20260. 23720740 10.1074/jbc.M113.455956 PMC3711292

[15] Zhang, J. , Clatterbuck, R. E. , Rigamonti, D. , Chang, D. D. and Dietz, H. C. , Interaction between krit1 and icap1alpha infers perturbation of integrin beta1‐mediated angiogenesis in the pathogenesis of cerebral cavernous malformation. Hum. Mol. Genet. 2001. 10: 2953–2960. 11741838 10.1093/hmg/10.25.2953

[16] Liu, W. , Draheim, K. M. , Zhang, R. , Calderwood, D. A. and Boggon, T. J. , Mechanism for KRIT1 release of ICAP1‐mediated suppression of integrin activation. Mol. Cell 2013. 49: 719–729. 23317506 10.1016/j.molcel.2012.12.005 PMC3684052

[17] Faurobert, E. , Rome, C. , Lisowska, J. , Manet‐Dupe, S. , Boulday, G. , Malbouyres, M. , Balland, M. et al., CCM1‐ICAP‐1 complex controls β integrin‐dependent endothelial contractility and fibronectin remodeling. J. Cell Biol. 2013. 202: 545–561. 23918940 10.1083/jcb.201303044 PMC3734079

[18] Bouvard, D. , Aszodi, A. , Kostka, G. , Block, M. R. , Albiges‐Rizo, C. and Fassler, R. , Defective osteoblast function in ICAP‐1‐deficient mice. Development 2007. 134: 2615–2625. 17567669 10.1242/dev.000877 PMC2793408

[19] Martinez‐Moreno, M. , Leiva, M. , Aguilera‐Montilla, N. , Sevilla‐Movilla, S. , Isern de Val, S. , Arellano‐Sanchez, N. , Gutierrez, N. C. et al., In vivo adhesion of malignant B cells to bone marrow microvasculature is regulated by α4β1 cytoplasmic‐binding proteins. Leukemia 2016. 30: 861–872. 26658839 10.1038/leu.2015.332

[20] Ruppert, R. , Moser, M. , Sperandio, M. , Rognoni, E. , Orban, M. , Liu, W. H. , Schulz, A. S. et al., Kindlin‐3‐mediated integrin adhesion is dispensable for quiescent but essential for activated hematopoietic stem cells. J. Exp. Med. 2015. 212: 1415–1432. 26282877 10.1084/jem.20150269 PMC4548061

[21] Papayannopoulou, T. , Craddock, C. , Nakamoto, B. , Priestley, G. V. and Wolf, N. S. , The VLA4/VCAM‐1 adhesion pathway defines contrasting mechanisms of lodgement of transplanted murine hemopoietic progenitors between bone marrow and spleen. Proc. Natl. Acad. Sci. U S A 1995. 92: 9647–9651. 7568190 10.1073/pnas.92.21.9647 PMC40859

[22] Leuker, C. E. , Labow, M. , Müller, W. and Wagner, N. , Neonatally induced inactivation of the vascular cell adhesion molecule 1 gene impairs B cell localization and T cell‐dependent humoral immune response. J. Exp. Med. 2001. 193: 755–768. 11257141 10.1084/jem.193.6.755 PMC2193422

[23] Rose, D. M. , Han, J. and Ginsberg, M. H. , Alpha4 integrins and the immune response. Immunol. Rev. 2002. 186: 118–124. 12234367 10.1034/j.1600-065x.2002.18611.x

[24] Chen, J. Y. , Miyanishi, M. , Wang, S. K. , Yamazaki, S. , Sinha, R. , Kao, K. S. , Seita, J. et al., Hoxb5 marks long‐term haematopoietic stem cells and reveals a homogenous perivascular niche. Nature 2016. 530: 223–227. 26863982 10.1038/nature16943 PMC4854608

[25] Xiong, J. , Armato, M. A. and Yankee, T. M. , Immature single‐positive CD8+ thymocytes represent the transition from Notch‐dependent to Notch‐independent T‐cell development. Int. Immunol. 2011. 23: 55–64. 21148236 10.1093/intimm/dxq457 PMC3031305

[26] Hogquist, K. A. , Xing, Y. , Hsu, F. C. and Shapiro, V. S. , T cell adolescence: maturation events beyond positive selection. J. Immunol. 2015. 195: 1351–1357. 26254267 10.4049/jimmunol.1501050 PMC4530466

[27] Xing, Y. , Wang, X. , Jameson, S. C. and Hogquist, K. A. , Late stages of T cell maturation in the thymus involve NF‐κB and tonic type I interferon signaling. Nat. Immunol. 2016. 17: 565–573. 27043411 10.1038/ni.3419 PMC4837029

[28] Ngo, V. N. , Tang, H. L. and Cyster, J. G. , Epstein‐Barr virus‐induced molecule 1 ligand chemokine is expressed by dendritic cells in lymphoid tissues and strongly attracts naive T cells and activated B cells. J. Exp. Med. 1998. 188: 181–191. 9653094 10.1084/jem.188.1.181 PMC2525549

[29] Ueno, T. , Saito, F. , Gray, D. H. , Kuse, S. , Hieshima, K. , Nakano, H. , Kakiuchi, T. et al., CCR7 signals are essential for cortex‐medulla migration of developing thymocytes. J. Exp. Med. 2004. 200: 493–505. 15302902 10.1084/jem.20040643 PMC2211934

[30] Yin, X. , Ladi, E. , Chan, S. W. , Li, O. , Killeen, N. , Kappes, D. J. and Robey, E. A. , CCR7 expression in developing thymocytes is linked to the CD4 versus CD8 lineage decision. J. Immunol. 2007. 179: 7358–7364. 18025179 10.4049/jimmunol.179.11.7358

[31] Fu, G. , Vallée, S. , Rybakin, V. , McGuire, M. V. , Ampudia, J. , Brockmeyer, C. , Salek, M. et al., Themis controls thymocyte selection through regulation of T cell antigen receptor‐mediated signaling. Nat. Immunol. 2009. 10: 848–856. 19597499 10.1038/ni.1766 PMC2757056

[32] Salomon, D. R. , Crisa, L. , Mojcik, C. F. , Ishii, J. K. , Klier, G. and Shevach, E. M. , Vascular cell adhesion molecule‐1 is expressed by cortical thymic epithelial cells and mediates thymocyte adhesion. Implications for the function of alpha4beta1 (VLA4) integrin in T‐cell development. Blood 1997. 89: 2461–2471. 9116290

[33] Prockop, S. E. , Palencia, S. , Ryan, C. M. , Gordon, K. , Gray, D. and Petrie, H. T. , Stromal cells provide the matrix for migration of early lymphoid progenitors through the thymic cortex. J. Immunol. 2002. 169: 4354–4361. 12370368 10.4049/jimmunol.169.8.4354

[34] Arroyo, A. G. , Yang, J. T. , Rayburn, H. and Hynes, R. O. , Differential requirements for alpha4 integrins during fetal and adult hematopoiesis. Cell 1996. 85: 997–1008. 8674127 10.1016/s0092-8674(00)81301-x

[35] Bungartz, G. , Stiller, S. , Bauer, M. , Muller, W. , Schippers, A. , Wagner, N. , Fassler, R. and Brakebusch, C. , Adult murine hematopoiesis can proceed without β1 and β7 integrins. Blood 2006. 108: 1857–1864. 16735603 10.1182/blood-2005-10-007658

[36] Gribi, R. , Hook, L. , Ure, J. and Medvinsky, A. , The differentiation program of embryonic definitive hematopoietic stem cells is largely alpha4 integrin independent. Blood 2006. 108: 501–509. 16551970 10.1182/blood-2005-10-4209

[37] Taniuchi, I. , Osato, M. , Egawa, T. , Sunshine, M. J. , Bae, S. C. , Komori, T. , Ito, Y. and Littman, D. R. , Differential requirements for Runx proteins in CD4 repression and epigenetic silencing during T lymphocyte development. Cell 2002. 111: 621–633. 12464175 10.1016/s0092-8674(02)01111-x

[38] Park, J. H. , Adoro, S. , Guinter, T. , Erman, B. , Alag, A. S. , Catalfamo, M. , Kimura, M. Y. et al., Signaling by intrathymic cytokines, not T cell antigen receptors, specifies CD8 lineage choice and promotes the differentiation of cytotoxic‐lineage T cells. Nat. Immunol. 2010. 11: 257–264. 20118929 10.1038/ni.1840 PMC3555225

[39] Egawa, T. , Tillman, R. E. , Naoe, Y. , Taniuchi, I. and Littman, D. R. , The role of the Runx transcription factors in thymocyte differentiation and in homeostasis of naive T cells. J. Exp. Med. 2007. 204: 1945–1957. 17646406 10.1084/jem.20070133 PMC2118679

[40] Woolf, E. , Xiao, C. , Fainaru, O. , Lotem, J. , Rosen, D. , Negreanu, V. , Bernstein, Y. et al., Runx3 and Runx1 are required for CD8 T cell development during thymopoiesis. Proc. Natl. Acad. Sci. U S A 2003. 100: 7731–7736. 12796513 10.1073/pnas.1232420100 PMC164656

[41] Bangsow, C. , Rubins, N. , Glusman, G. , Bernstein, Y. , Negreanu, V. , Goldenberg, D. , Lotem, J. et al., The RUNX3 gene—sequence, structure and regulated expression. Gene 2001. 279: 221–232. 11733147 10.1016/s0378-1119(01)00760-0

[42] He, X. , Dave, V. P. , Zhang, Y. , Hua, X. , Nicolas, E. , Xu, W. , Roe, B. A. and Kappes, D. J. , The zinc finger transcription factor Th‐POK regulates CD4 versus CD8 T‐cell lineage commitment. Nature 2005. 433: 826–833. 15729333 10.1038/nature03338

[43] Singer, A. , Adoro, S. and Park, J. H. , Lineage fate and intense debate: myths, models and mechanisms of CD4‐ versus CD8‐lineage choice. Nat. Rev. Immunol. 2008. 8: 788–801. 18802443 10.1038/nri2416 PMC2760737

[44] Luckey, M. A. , Kimura, M. Y. , Waickman, A. T. , Feigenbaum, L. , Singer, A. and Park, J. H. , The transcription factor ThPOK suppresses Runx3 and imposes CD4(+) lineage fate by inducing the SOCS suppressors of cytokine signaling. Nat. Immunol. 2014. 15: 638–645. 24880459 10.1038/ni.2917 PMC6693509

[45] Hough, M. R. , Takei, F. , Humphries, R. K. and Kay, R. , Defective development of thymocytes overexpressing the costimulatory molecule, heat‐stable antigen. J. Exp. Med. 1994. 179: 177–184. 8270863 10.1084/jem.179.1.177 PMC2191310

[46] Pillai, S. and Cariappa, A. , The follicular versus marginal zone B lymphocyte cell fate decision. Nat. Rev. Immunol. 2009. 9: 767–777. 19855403 10.1038/nri2656

[47] Arnon, T. I. , Horton, R. M. , Grigorova, I. L. and Cyster, J. G. , Visualization of splenic marginal zone B‐cell shuttling and follicular B‐cell egress. Nature 2013. 493: 684–688. 23263181 10.1038/nature11738 PMC3561487

[48] Lenter, M. , Uhlig, H. , Hamann, A. , Jeno, P. , Imhof, B. and Vestweber, D. , A monoclonal antibody against an activation epitope on mouse integrin chain beta 1 blocks adhesion of lymphocytes to the endothelial integrin alpha 6 beta 1. Proc. Natl. Acad. Sci. U S A 1993. 90: 9051–9055. 7692444 10.1073/pnas.90.19.9051 PMC47499

[49] Shah, D. K. and Zuniga‐Pflucker, J. C. , An overview of the intrathymic intricacies of T cell development. J. Immunol. 2014. 192: 4017–4023. 24748636 10.4049/jimmunol.1302259

[50] Gascoigne, N. R. , Rybakin, V. , Acuto, O. and Brzostek, J. , TCR signal strength and T cell development. Annu. Rev. Cell Dev. Biol. 2016. 32: 327–348. 27712102 10.1146/annurev-cellbio-111315-125324

[51] Levanon, D. and Groner, Y. , Structure and regulated expression of mammalian RUNX genes. Oncogene 2004. 23: 4211–4219. 15156175 10.1038/sj.onc.1207670

[52] Surh, C. D. , Ernst, B. and Sprent, J. , Growth of epithelial cells in the thymic medulla is under the control of mature T cells. J. Exp. Med. 1992. 176: 611–616. 1500862 10.1084/jem.176.2.611 PMC2119324

[53] Gray, D. H. , Seach, N. , Ueno, T. , Milton, M. K. , Liston, A. , Lew, A. M. , Goodnow, C. C. and Boyd, R. L. , Developmental kinetics, turnover, and stimulatory capacity of thymic epithelial cells. Blood 2006. 108: 3777–3785. 16896157 10.1182/blood-2006-02-004531

[54] Saini, M. , Sinclair, C. , Marshall, D. , Tolaini, M. , Sakaguchi, S. and Seddon, B. , Regulation of Zap70 expression during thymocyte development enables temporal separation of CD4 and CD8 repertoire selection at different signaling thresholds. Sci. Signal 2010. 3: ra23. 20332428 10.1126/scisignal.2000702

[55] Lu, T. T. and Cyster, J. G. , Integrin‐mediated long‐term B cell retention in the splenic marginal zone. Science 2002. 297: 409–412. 12130787 10.1126/science.1071632

[56] Saito, T. , Chiba, S. , Ichikawa, M. , Kunisato, A. , Asai, T. , Shimizu, K. , Yamaguchi, T. et al., Notch2 is preferentially expressed in mature B cells and indispensable for marginal zone B lineage development. Immunity 2003. 18: 675–685. 12753744 10.1016/s1074-7613(03)00111-0

[57] Tanigaki, K. , Han, H. , Yamamoto, N. , Tashiro, K. , Ikegawa, M. , Kuroda, K. , Suzuki, A. et al., Notch‐RBP‐J signaling is involved in cell fate determination of marginal zone B cells. Nat. Immunol. 2002. 3: 443–450. 11967543 10.1038/ni793

[58] Kaisho, T. , Takeda, K. , Tsujimura, T. , Kawai, T. , Nomura, F. , Terada, N. and Akira, S. , IkappaB kinase alpha is essential for mature B cell development and function. J. Exp. Med. 2001. 193: 417–426. 11181694 10.1084/jem.193.4.417 PMC2195900

[59] Cariappa, A. , Liou, H. C. , Horwitz, B. H. and Pillai, S. , Nuclear factor kappa B is required for the development of marginal zone B lymphocytes. J. Exp. Med. 2000. 192: 1175–1182. 11034607 10.1084/jem.192.8.1175 PMC2195875

[60] Montero‐Herradón, S. , García‐Ceca, J. and Zapata, A. G. , Altered maturation of medullary TEC in EphB‐deficient thymi is recovered by RANK signaling stimulation. Front Immunol. 2018. 9: 1020. 29867988 10.3389/fimmu.2018.01020 PMC5954084

[61] Garcia‐Bernal, D. , Sotillo‐Mallo, E. , Nombela‐Arrieta, C. , Samaniego, R. , Fukui, Y. , Stein, J. V. and Teixido, J. , DOCK2 is required for chemokine‐promoted human T lymphocyte adhesion under shear stress mediated by the integrin alpha4beta1. J. Immunol. 2006. 177: 5215–5225. 17015707 10.4049/jimmunol.177.8.5215

[62] Sevilla‐Movilla, S. , Arellano‐Sánchez, N. , Martínez‐Moreno, M. , Gajate, C. , Sánchez‐Vencells, A. , Valcárcel, L. V. , Agirre, X. et al., Upregulated expression and function of the α4β1 integrin in multiple myeloma cells resistant to bortezomib. J. Pathol. 2020. 252: 29–40. 32501543 10.1002/path.5480

[63] Barrio, L. , Román‐García, S. , Díaz‐Mora, E. , Risco, A. , Jiménez‐Saiz, R. , Carrasco, Y. R. and Cuenda, A. , B cell development and T‐dependent antibody response are regulated by p38γ and p38δ. Front Cell Dev. Biol. 2020. 8: 189. 32266269 10.3389/fcell.2020.00189 PMC7105866

