# Supplementary material for: ICAP‐1 loss impairs CD8+ thymocyte development and leads to reduced marginal zone B cells in mice
Source: Eur J Immunol. 2022 May 13;52(8):1228–42. doi: 10.1002/eji.202149560 (PMC9543158; doi:10.1002/eji.202149560)
Supplement: Supplementary file 1 — Fig. S1. Bone marrow and thymic properties of ICAP‐1‐null mice Fig. S2. Analysis of thymocyte subpopulations in ICAP‐1‐deficient mice Fig. S3. Characterization of thymic epithelial cells, thymus architecture, and analysis of BM chimerae Fig. S4. Expression of b1 (CD29) in thymocyte subsets from ICAP‐1‐deficient mice Fig. S5. Spleen and lymph node cell numbers, gating strategies and functional assessment of spleen T cells. Fig. S6. Spleen and lymph node B cell distribution and B cell proliferation analyses Fig. S7. Expression of b1, talin and kindlin‐3 in spleen cells from ICAP‐1‐deficient mice Table S1. List of antibodies used for flow cytometry Table S2. Oligonucleotide sequences used for qRT‐PCR assays [file EJI-52-1228-s001.pdf]

SUPPORTING INFORMATION

MATERIALS AND METHODS

Tissue immunofluorescence. For spleen immunofluorescence we followed the described method (1). Briefly, spleen sections were stained with FITC-conjugated rat anti-mouse IgD (BD Bioscience), Cy5-goat anti-mouse IgM (Jackson Immunoresearch, Philadelphia, PA) and biotin rat anti-mouse CD169/MOMA-1 antibody (Abcam, Cambridge, UK) plus Alexa 568 streptavidin (Molecular Probes). Sections were mounted in Fluoromount and imaged on a Leica TCS-SP5 confocal microscope. Quantification of the MZ thickness was carried out using the Fiji (ImageJ) software. For this, measurements of the thickness of the MZ in different regions of five different ICAP$^{+/+}$ and ICAP$^{-/-}$ spleen follicles were performed, and subsequently the means were obtained. Thymic frozen sections were fixed in acetone, and cryosections stained with antibodies for TECs: rabbit anti-mouse K5 (AF138; Covance, Princeton, NJ), and FITC-anti-cytokeratin pan Ab (C11: Sigma Aldrich), or for thymocytes (CD4 and CD8). Labeling was detected with Alexa Fluor 405-conjugated goat anti-rabbit IgG (Thermo Fisher Scientific). Finally, sections were mounted with SlowFade™ Diamond (Thermo Fisher Scientific) and imaged on a Leica SP2 confocal microscope. All images were analyzed using LasX software.

Real-time quantitative PCR. RT-PCR of TECs was carried out using SSIII Platinum Taq Mix (Invitrogen). RT-PCR of selected genes was performed in triplicate by using TaqMan-probe assays in an AB fast-7900HT System (Thermo Fisher) under standard running conditions. Predesigned TaqMan assays for target genes were: Mm00492707_m1 *(ITGB1BP1)* and Mm00493214_m1 (*EpCAM*). The TaqMan assay for endogenous gene was Mm99999915_g1 (*GAPDH*). Ct assignment was performed using the Sequence Detection System 2.4 software (Applied Biosystems) to set baseline and threshold

parameters. Assay efficiency was assumed to be 2. Expression levels of target genes in treated cells were normalized to expression of *GAPDH* and results were referred to Control samples. Relative changes and statistical values of mRNA expression levels were calculated by the ddCt method.

REFERENCES

1. L. Barrio *et al.*, B Cell Development and T-Dependent Antibody Response Are Regulated by p38γ and p38δ. *Front Cell Dev Biol* **8**, 189 (2020).

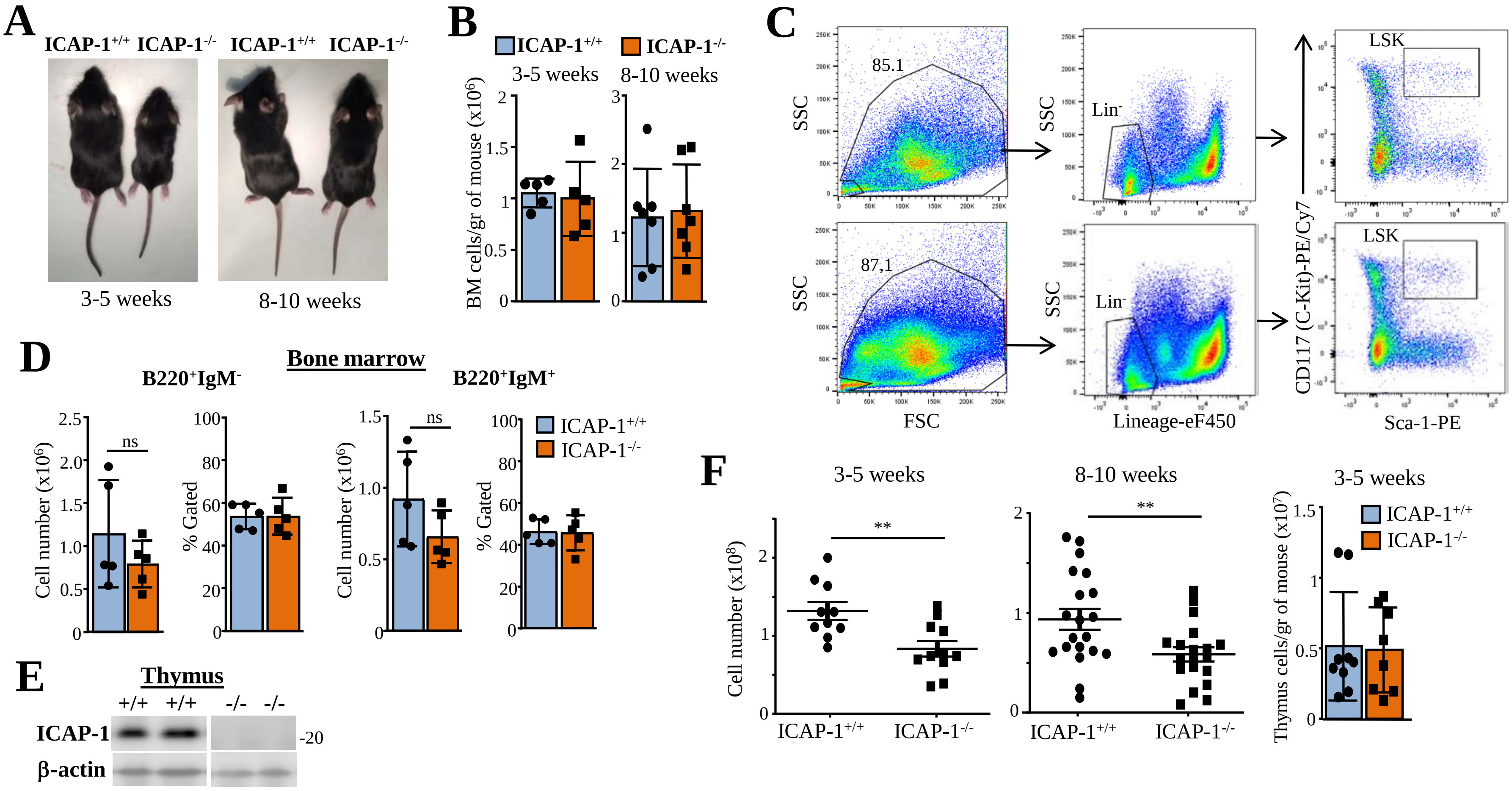

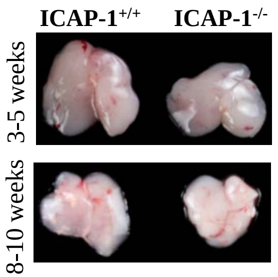

Fig. S1. Bone marrow and thymic properties of ICAP-1-null mice. (A) Gross appearance of ICAP-1$^{+/+}$ and ICAP-1$^{-/-}$ mice at different ages. (B) BM cell numbers from femur and tibia relative to body weight. (C) Gating strategy for LSK bone marrow cell populations (see Fig. 1A). (D) Cell number and proportions of the B220^{+}IgM^{-} and B220^{+}IgM^{+} fractions were analyzed by flow cytometry. (E, top) Expression of thymic ICAP-1 was analyzed by immunoblotting . (Bottom) Pictures depict control and ICAP-1-deficient thymi. (F, left and middle) Total cellularity of control and ICAP-1$^{-/-}$ thymi at the indicated ages ($^{**}p<0.01$). (Right) Thymus cell numbers relative to body weight. Panels B, D and F show pooled data from 2-6 independent experiments.

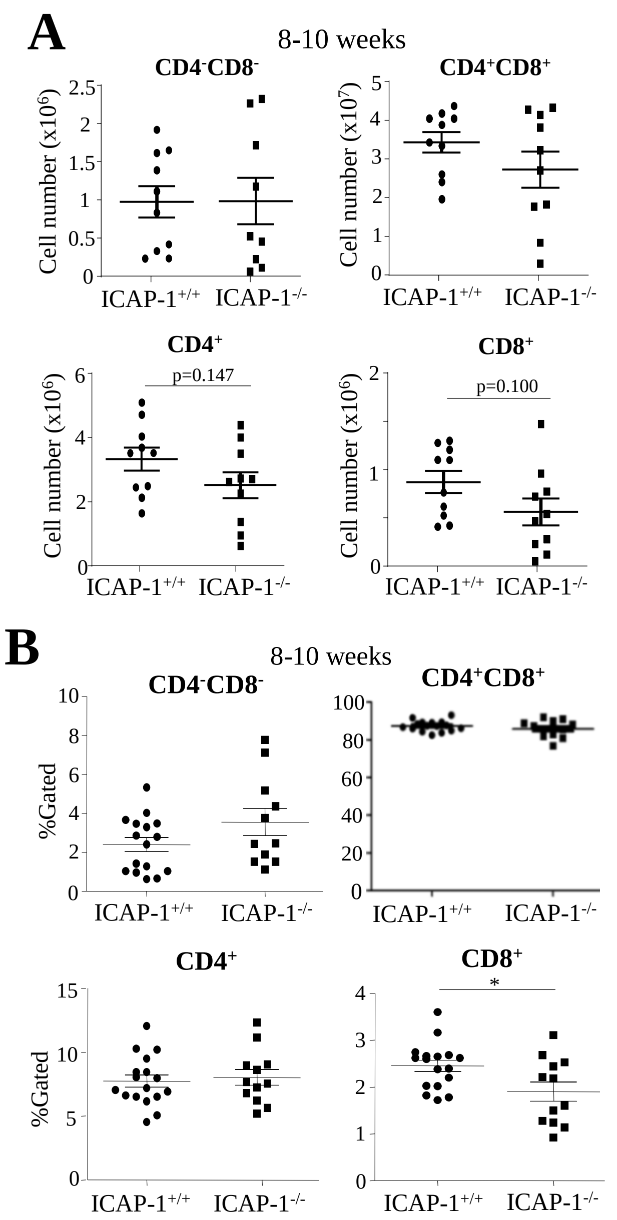

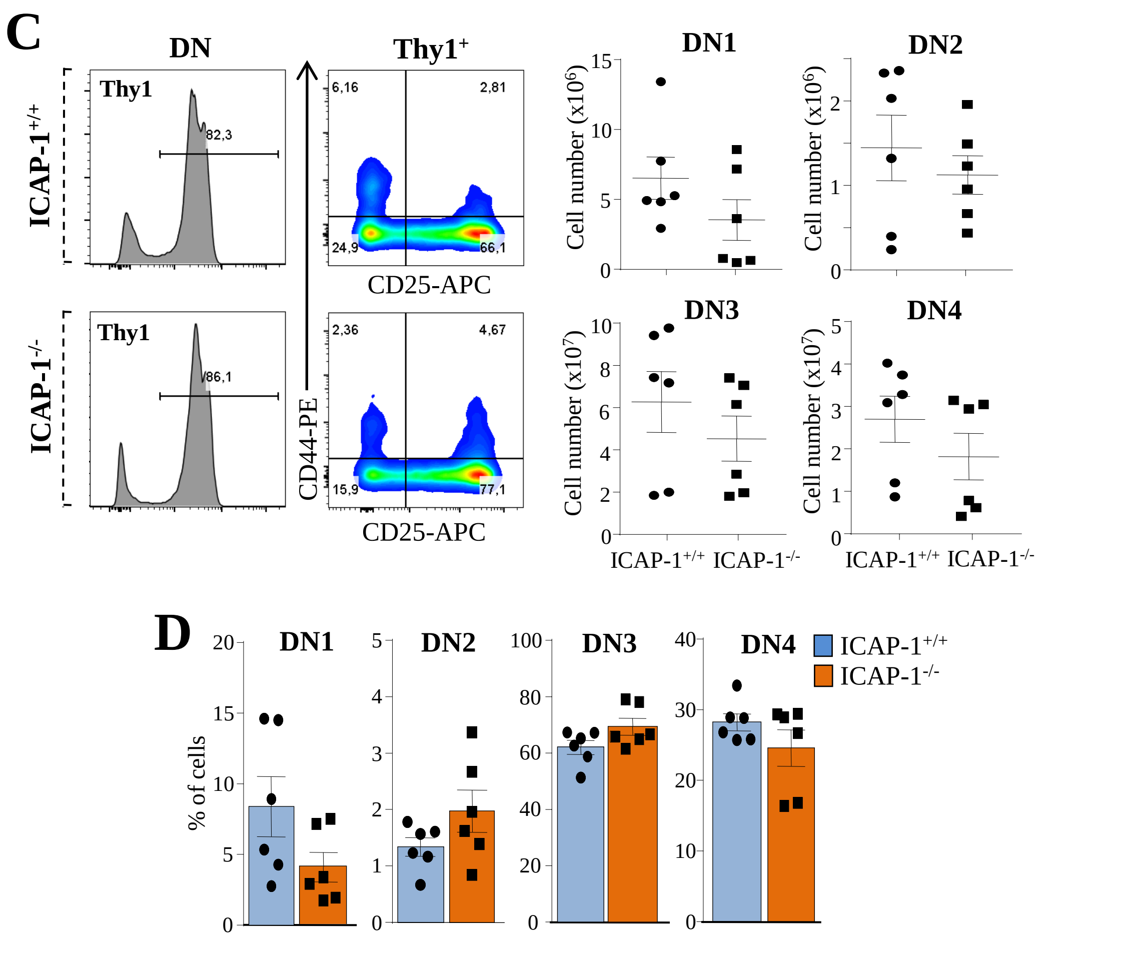

Fig. S2. Analysis of thymocyte subpopulations in ICAP-1-deficient mice. Total numbers (A) and proportions (B) of the indicated thymocyte subsets in ICAP-$1^{+/+}$ and ICAP-$1^{-/-}$ mice were analyzed by flow cytometry ($^{*}p<0.05$). $CD4^{-}CD8^{-}$ double negative (DN) cells were gated with a Thy1-BV421 antibody, and the total cell numbers (C) and distribution (D) of the DN1, DN2, DN3 and DN4 subsets in the Thy^{+} cells was assessed by flow cytometry. A-D panels show pooled data from at least three independent experiments.

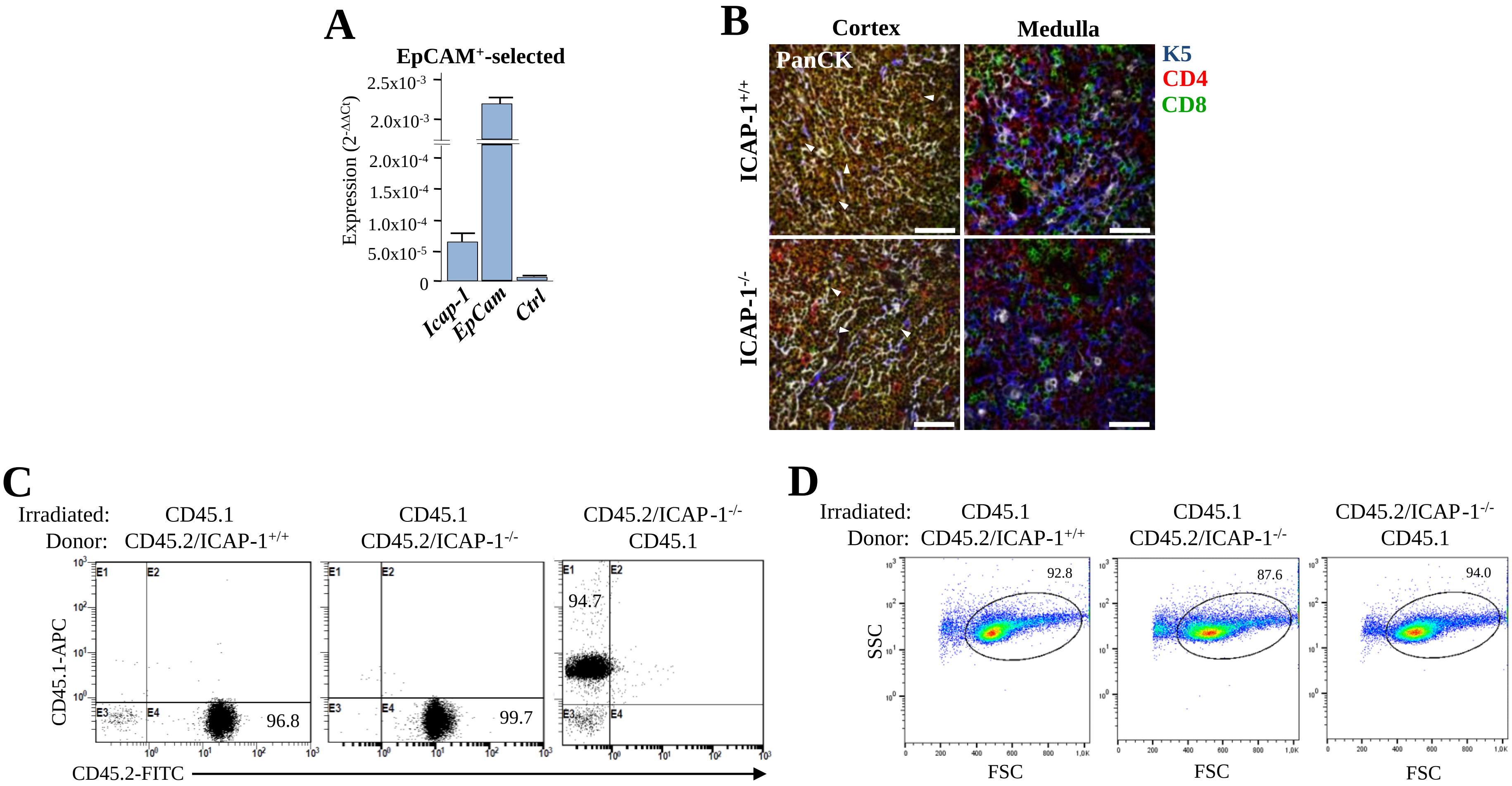

Fig. S3. Characterization of thymic epithelial cells, thymus architecture, and analysis of BM chimerae. (A) Expression of *Icap-1* in EpCAM-1-selected TECs was assessed by qPCR. (B) Confocal microscopy of ICAP-1$^{+/+}$ and ICAP-1$^{-/-}$ thymi sections showing the PanCK^{+} thymic cortex and the medullary K5^{+} (blue) area. DP thymocytes (arrowheads) in the cortex, and CD4^{+} (red) or CD8^{+} (green) thymocytes in the medulla are shown. Scale bar: 50 µm. (C) Representative CD45.1 and CD45.2 FACS dot plots of thymi from the indicated BM chimeras. (D) FSC/SSC profiles of thymic samples from the BM chimeras that were subsequently analyzed for the proportions of CD4^{+} and CD8^{+} cells (see Fig. 2F).

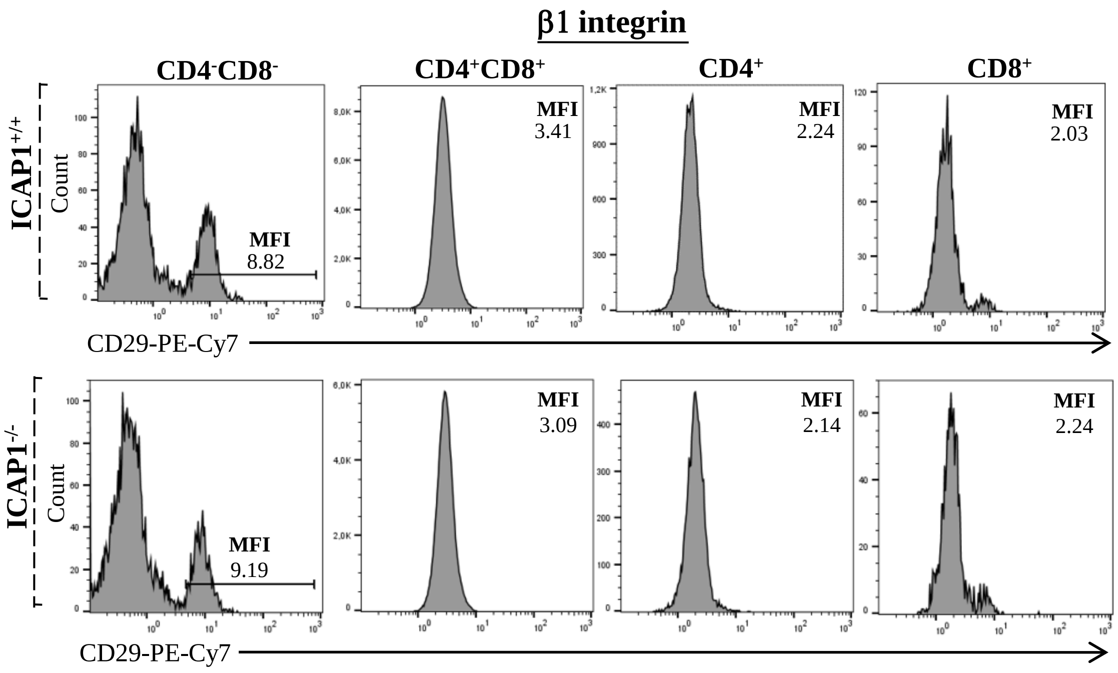

Fig. S4. Expression of β1 (CD29) in thymocyte subsets from ICAP-1-deficient mice. The indicated thymocyte subpopulations from control and ICAP-1-null thymi were gated as shown in Fig. 2B, and the expression of β1 integrin assessed by flow cytometry. Shown is a representative result out of three independent experiments. (MFI, mean fluorescence intensity).

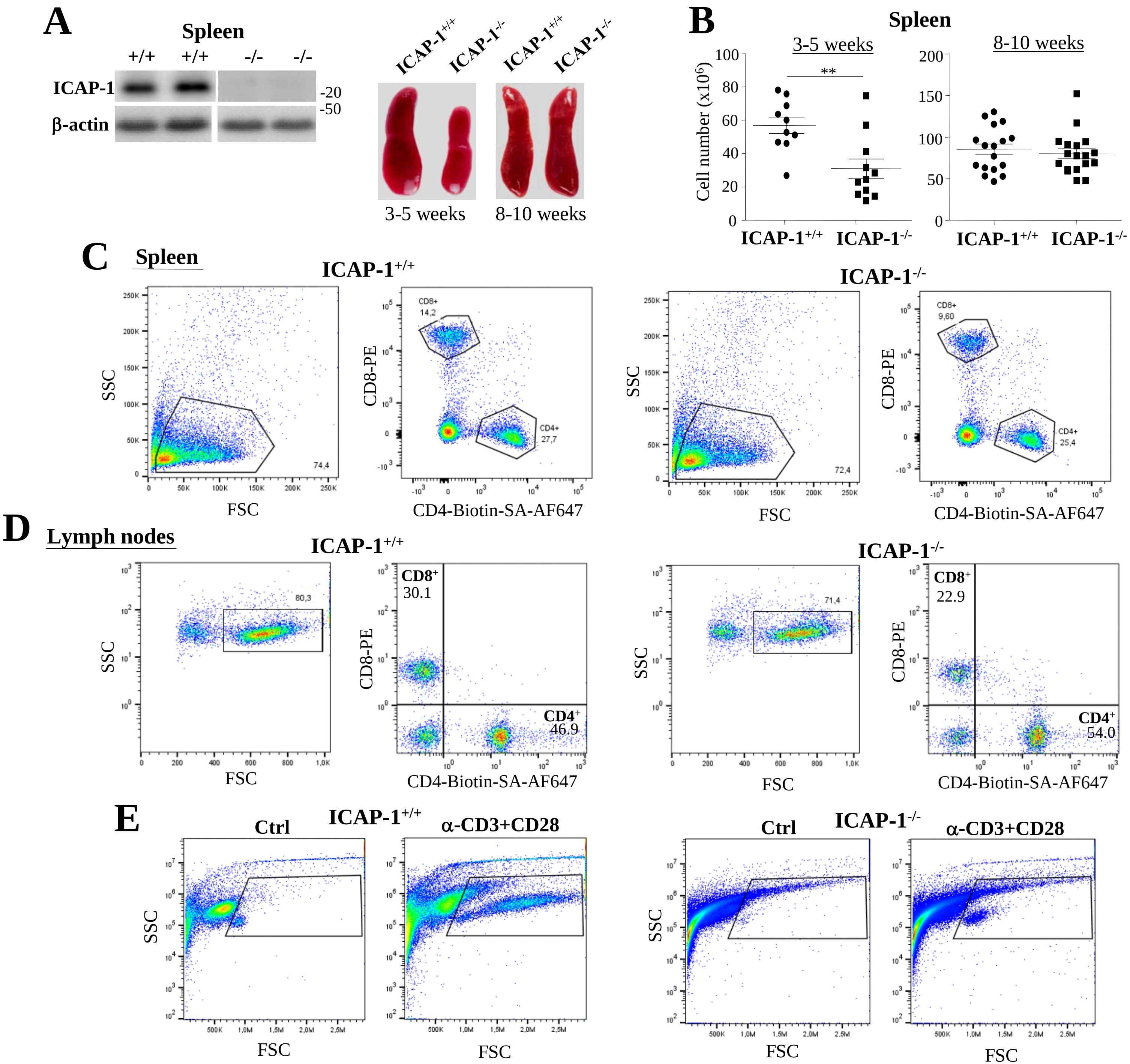
A
Spleen
+/+ +/+ -/- -/-
ICAP-1
β-actin
-20
-50
ICAP-1+/+ ICAP-1-/- ICAP-1+/+ ICAP-1-/-
3-5 weeks 8-10 weeks
B
Spleen
3-5 weeks
8-10 weeks
Cell number (x10⁶)
**
ICAP-1+/+ ICAP-1-/- ICAP-1+/+ ICAP-1-/-
C
Spleen
ICAP-1+/+
ICAP-1-/-
SSC
FSC
CD8-PE
CD4-Biotin-SA-AF647
CD8+ 14,2
CD4+ 27,7
74,4
CD8+ 9,60
CD4+ 25,4
72,4
D
Lymph nodes
ICAP-1+/+
ICAP-1-/-
80,3
CD8+ 30.1
CD4+ 46.9
71,4
CD8+ 22.9
CD4+ 54.0
E
ICAP-1+/+
ICAP-1-/-
Ctrl
α-CD3+CD28

Fig. S5. Spleen and lymph node cell numbers, gating strategies and functional assessment of spleen T cells. (A, left) ICAP-1 expression in spleens was analyzed by immunoblotting. Shown is a representative result out of three independent experiments. (Right) Shown are representative spleens from control and ICAP-1$^{-/-}$ animals. (B) Total cellularity of spleens from ICAP-1$^{-/-}$ mice relative to control mice ($^{**}p<0.01$). (C, D) FSC/SSC-gated total spleen and lymph node lymphocytes were analyzed for frequencies of CD4^{+} and CD8^{+} T cells. Shown is a representative result out of 3-5 independent experiments (see Fig. 5B). (E) CD3^{+}-selected spleen T cells from control and ICAP-1-deficient mice were labeled with CellTracer Violet and incubated in the absence (Ctrl) or presence of co-immobilized anti-CD3 and anti-CD28 antibodies. Depicted are the flow cytometry FSC/SSC patterns of a representative result out of two independent experiments.

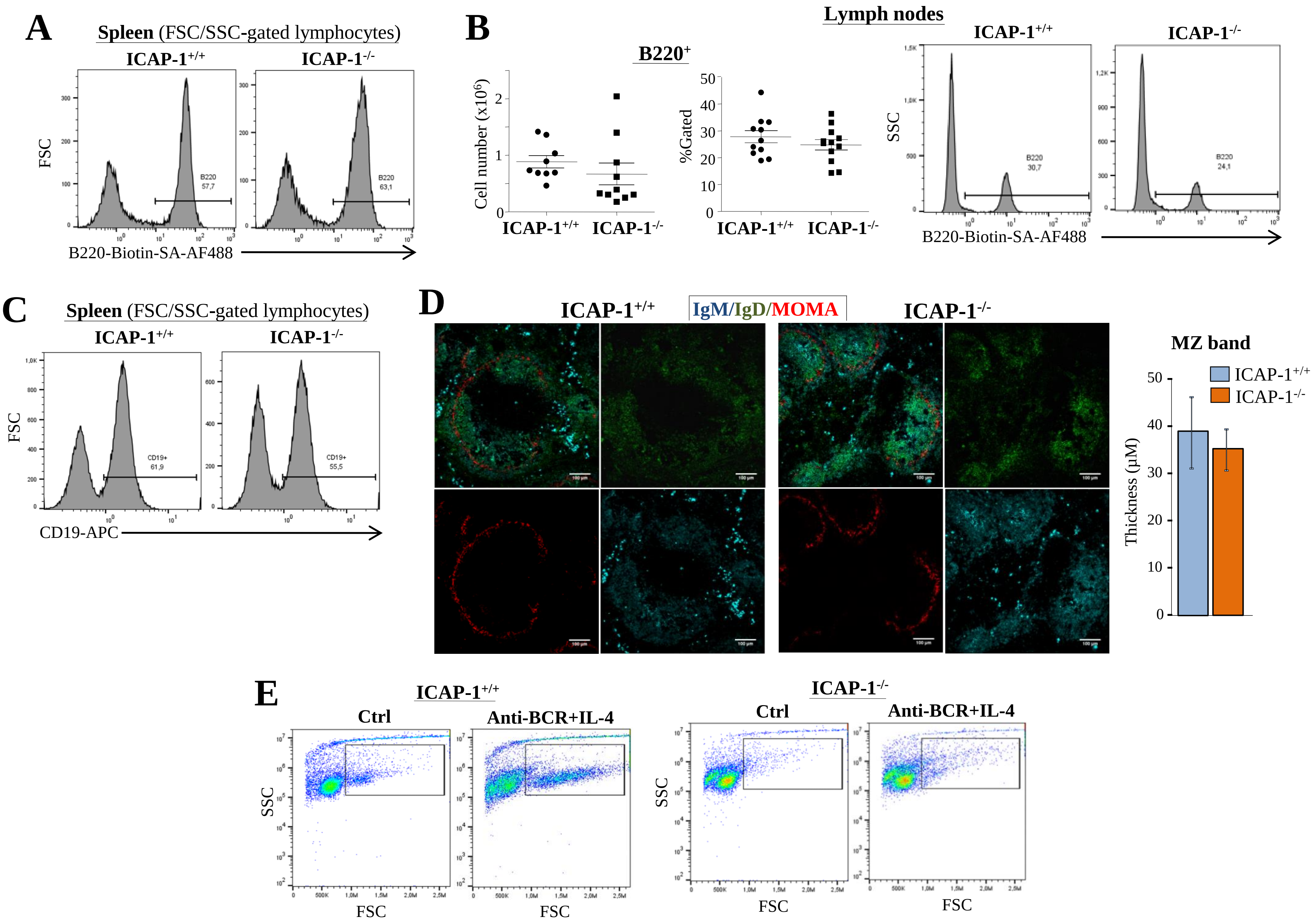

A
Spleen (FSC/SSC-gated lymphocytes)
ICAP-1+/+
ICAP-1-/-
FSC
B220 57,7
B220 63,1
B220-Biotin-SA-AF488
B
B220+
Cell number (x10^6)
%Gated
ICAP-1+/+
ICAP-1-/-
Lymph nodes
SSC
B220 30,7
B220 24,1
B220-Biotin-SA-AF488
C
Spleen (FSC/SSC-gated lymphocytes)
ICAP-1+/+
ICAP-1-/-
FSC
CD19+ 61,9
CD19+ 55,5
CD19-APC
D
ICAP-1+/+
IgM/IgD/MOMA
ICAP-1-/-
MZ band
ICAP-1+/+
ICAP-1-/-
Thickness (µM)
E
ICAP-1+/+
Ctrl
Anti-BCR+IL-4
ICAP-1-/-
Ctrl
Anti-BCR+IL-4
SSC
FSC

Fig. S6. Spleen and lymph node B cell distribution and B cell proliferation analyses. (A, B) Shown are histograms of FSC/SSC-gated spleen and lymph node lymphocytes (see Figs. S5C and S5D) from control and ICAP-1$^{-/-}$ mice displaying B220^{+} cell proportions. Data on panel B right represent pooled data from three independent experiments. (C) Histograms of FSC/SSC-gated spleen lymphocytes showing CD19^{+} B cell proportions. Panels A-C are representative results out of at least three independent experiments. (D, left) Fluorescence images of IgM (blue), IgD (green) and MOMA-1 (red) expression in representative tissue sections from spleens of ICAP-1$^{+/+}$ and ICAP-1$^{-/-}$ mice (n=4 for each genotype). (Right) Shown is the quantification of MZ thickness. (E) CD19^{+}-selected spleen B cells from control and ICAP-1-deficient mice were labeled with CellTracer Violet and incubated in the absence (Ctrl) or presence of anti-BCR and IL-4. Depicted are the flow cytometry FSC/SSC patterns of a representative result out of two independent experiments.

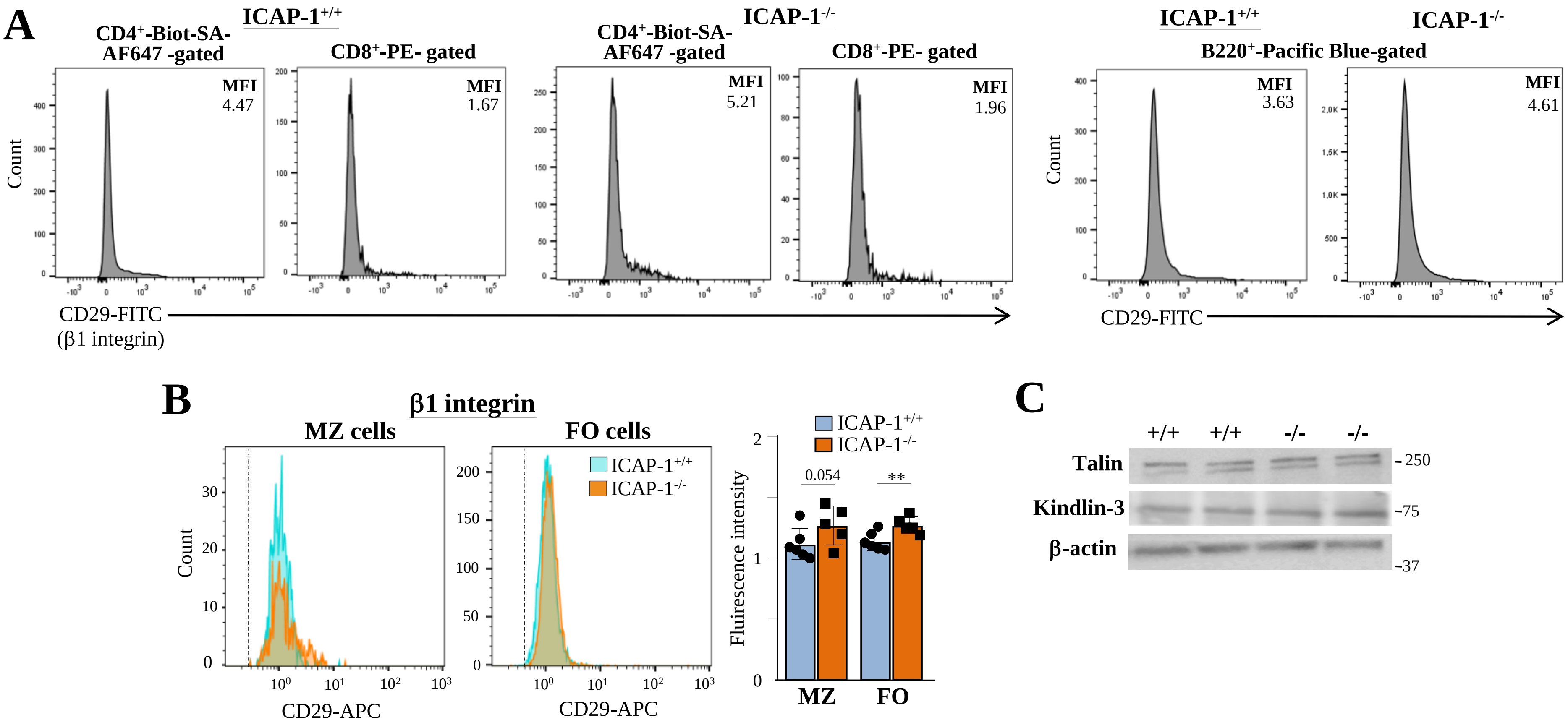

Fig. S7. Expression of β1, talin and kindlin-3 in spleen cells from ICAP-1-deficient mice. (A) Lymphocytes from control and ICAP-1-null spleens were gated as indicated and analyzed for the expression of β1 integrin. Shown is a representative result out of four independent experiments. (MFI, mean fluorescence intensity). (B) Analysis and quantification of β1 expression on MZ and FO spleen B cells. A representative histogram is shown (left), and right panel displays pooled data from two independent experiments. (C) Expression of talin and kindlin-3 in the spleens of control (+/+) and ICAP-1-null mice was determined by immunoblotting. Shown is a representative result out of four independent experiments.

Table S1. List of antibodies used for flow cytometry

ANTIBODY (Clone)	PROVIDER	ANTIBODY (Clone)	PROVIDER
FITC rat anti-mouse CD29 (HMβ1)	BioLegend	PerCP/Cy5.5 rat anti-mouse CD150 (SLAM) (TC15-12F12.2)	BioLegend
PE/Cy7 rat anti-mouse CD29 (HMβ1)	BioLegend	FITC Rat anti-mouse CD93 (AA4.1)	eBioscience
APC Armenian hamster anti-mouse/rat CD29 (HMβ1-1)	BioLegend	PerCP anti-mouse CD45 (30-F11)	BioLegend
Alexa Fluor® 647 Armenian hamster anti-mouse CD3ε (145-2C11)	BioLegend	PE-Cy5.5 rat anti-mouse/human CD45R (B220) (RA3-6B2)	eBioscience
PE/Cy7 Armenian hamster anti-mouse CD3	BD Bioscience	FITC rat anti-mouse CD45R/B220 (RA3-6B2)	Southern Biotech
BV421 rat anti-mouse CD8a (53-6.7)	BioLegend	PE rat anti-mouse CD45R/B220 (RA3-6B2)	Southern Biotech
PE rat anti-mouse CD8a (53-6.7)	eBioscience	Biotin rat anti-mouse/human CD45R/B220 (RA3-6B2)	BioLegend
FITC rat anti-mouse CD8a (53-6.7)	Southern Biotech	Pacific Blue rat anti-mouse/human CD45R/B220 (RA3-6B2)	BioLegend
FITC rat anti-mouse CD4 (GK1.5)	Southern Biotech	APC Mouse anti-mouse CD45.1 (A20)	Immunostep
Biotin rat anti-mouse CD4 (GK1.5)	Southern Biotech	FITC Mouse anti-mouse CD45.2 (104)	BD Pharmingen
PerCP-Cy5.5 rat anti-mouse CD4 (RM4-5)	BD Bioscience	PE rat anti-mouse CD19 (6D5)	Southern Biotech
Alexa Fluor 647 rat anti-mouse CD4 (GK1.5)	Biolegend	APC rat anti-mouse CD19 (1D3)	eBioscience
PE rat anti-mouse CD24 (M1/69)	BD Bioscience	Alexa Fluor 647 rat anti-mouse IgM (RMM-1)	BioLegend
APC rat anti-mouse CD25 (PC61.5)	eBioscience	FITC rat anti-mouse IgD (11-26)	eBioscience
PE/Cy7 rat anti-mouse CD25 (PC61.5)	BD Bioscience	Biotin rat anti-mouse CD43 (S7)	Pharmingen
PE rat anti-mouse CD44 (IM7)	BD Bioscience	FITC rat anti-mouse CD21/CD35 (4E3)	eBioscience
BV421 rat anti-mouse CD44 (IM7)	BioLegend	PE rat anti-mouse CD23 (B3B4)	eBioscience
Biotin anti-mouse CD62L (MEL-14)	BD Bioscience	FITC hamster anti-mouse CD69 (H1.2F3)	BD Bioscience
Pacific Blue-CD90.2(Thy1) rat anti-mouse (30-H12)	Biolegend	PE mouse anti-mouse H2-Kb (AF6-88.5)	BD Bioscience
PerCP-Cy5.5 rat anti-mouse CD197 (CCR7) (4B12)	eBioscience	FITC hamster anti-mouse TCRαβ (H57-597)	Immunotools
eFluor 450 mouse Hematopoietic Lineage Antibody	eBioscience	Alexa Fluor 488 rat anti-mouse CD326 (EpCAM) (G8.8)	BioLegend
PE Ly-6A/E (Sca-1) rat anti-mouse (D7)	eBioscience	PE rat anti-mouse Ly-51 (6C3)	BioLegend
PE-Cy7 rat anti-mouse/pig CD117 (2B8)	eBioscience	Rabbit anti-mouse K5 (AF138)	Covance
FITC rat anti-mouse CD34 (RAM34)	eBioscience	Pacific Blue-Streptavidin	Molecular Probes
APC Armenian hamster anti-mouse CD48 (HM48-1)	BioLegend	Alexa Fluor 488-Streptavidin	BioLegend
FITC-anti-cytokeratin pan Ab (C11)	Sigma Aldrich	Alexa Fluor 647-Streptavidin	BioLegend
Biotin-UEA-1	Vector Laboratories	Alexa Fluor 546-Streptavidin	Invitrogen
Biotin-H2Kb anti-mouse (AF6-88.5)	BioLegend	APC/Cy7-Streptavidin	BioLegend
		APC-Streptavidin	BioLegend

Table S2. Oligonucleotide sequences used for qRT-PCR assays

**Target**	**Forward (5'-3')**	**Reverse (5'-3')**
TBP	GGGGAGCTGTGATGTGAAGT	CCAGGAAATAATTCTGGCTCA
THPOK	GACTTGGTGGGAGCTTAACCC	TCCCCATCTTCTCACTTCCTGGT
SOCS1	CCGCCAGATGAGCCCAC	GGTTGCGTGCTACCATCCTA
RUNX3d	GCGACATGGCTTCCAACAGC	CTTAGCGCGCCGCTGTTCTCGC
GATA3	GCTCCTTGCTACTCAGGTGAT	GGAGGGAGAGAGGAATCCGA
ICAP-1	GCCTGTGGGTTTATCAGTGC	AAGCGGTGGATAAAACCTTG
